# Molecular mechanism of the arrestin-biased agonism of neurotensin receptor 1 by an intracellular allosteric modulator

**DOI:** 10.1038/s41422-025-01095-7

**Published:** 2025-03-21

**Authors:** Demeng Sun, Xiang Li, Qingning Yuan, Yuanxia Wang, Pan Shi, Huanhuan Zhang, Tao Wang, Wenjing Sun, Shenglong Ling, Yuanchun Liu, Jinglin Lai, Wenqin Xie, Wanchao Yin, Lei Liu, H. Eric Xu, Changlin Tian

**Affiliations:** 1 Hefei National Laboratory for Physical Sciences at the Microscale, Joint Center for Biological Analytical Chemistry, Division of Life Sciences and Medicine, University of Science and Technology of China, Hefei, Anhui China; 2 Tsinghua-Peking Center for Life Sciences, Ministry of Education Key Laboratory of Bioorganic Phosphorus Chemistry and Chemical Biology, Center for Synthetic and Systems Biology, Department of Chemistry, Tsinghua University, Beijing, China; 3 State Key Laboratory of Drug Research, Shanghai Advanced Electron Microscope Center, Shanghai Institute of Materia Medica, Chinese Academy of Sciences, Shanghai, China; 4 School of Biomedical Engineering and Suzhou Institute for Advanced Research, University of Science and Technology of China, Suzhou, Jiangsu China; 5 Zhongshan Institute for Drug Discovery, Shanghai Institute of Materia Medica, Chinese Academy of Sciences, Zhongshan, Guangdong China; 6 School of Pharmaceutical Sciences, Southern Medical University, Guangzhou, Guangdong China; 7 University of Chinese Academy of Sciences, Beijing, China; 8 School of Chemistry and Chemical Engineering & Zhangjiang Institute for Advanced Study, Shanghai Jiao Tong University, Shanghai, China

**Keywords:** Cryoelectron microscopy, Hormone receptors

## Abstract

Biased allosteric modulators (BAMs) of G protein-coupled receptors (GPCRs) have been at the forefront of drug discovery owing to their potential to selectively stimulate therapeutically relevant signaling and avoid on-target side effects. Although structures of GPCRs in complex with G protein or GRK in a BAM-bound state have recently been resolved, revealing that BAM can induce biased signaling by directly modulating interactions between GPCRs and these two transducers, no BAM-bound GPCR–arrestin complex structure has yet been determined, limiting our understanding of the full pharmacological profile of BAMs. Herein, we developed a chemical protein synthesis strategy to generate neurotensin receptor 1 (NTSR1) with defined hexa-phosphorylation at its C-terminus and resolved high-resolution cryo-EM structures (2.65–2.88 Å) of NTSR1 in complex with both β-arrestin1 and the BAM SBI-553. These structures revealed a unique “loop engagement” configuration of β-arrestin1 coupling to NTSR1 in the presence of SBI-553, markedly different from the typical “core engagement” configuration observed in the absence of BAMs. This configuration is characterized by the engagement of the intracellular loop 3 of NTSR1 with a cavity in the central crest of β-arrestin1, representing a previously unobserved, arrestin-selective conformation of GPCR. Our findings fill the critical knowledge gap regarding the regulation of GPCR–arrestin interactions and biased signaling by BAMs, which would advance the development of safer and more efficacious GPCR-targeted therapeutics.

## Introduction

G protein-coupled receptors (GPCRs) constitute the most abundant class of cell surface receptors in the human genome and are the most prolific targets for FDA-approved drugs in the treatment of a broad-spectrum conditions ranging from pain, diabetes, cardiovascular disease to cancer.^1–3^ GPCRs pose challenges for drug discovery efforts particularly in achieving receptor subtype selectivity and controlling on- and off-target side effects, which are not always possible with classic orthosteric ligands.^4–8^ The development of allosteric modulators that engage less well-conserved regulatory motifs outside the orthosteric pocket has emerged as a promising avenue to address these challenges.^3,7,9^ Unlike conventional orthosteric ligands, which either continuously activate or inhibit signaling, allosteric modulators possess the ability to either promote (acting as positive allosteric modulator, PAM) or suppress (acting as negative allosteric modulator, NAM) the signaling responses to endogenous ligands,^3,10^ while also providing excellent receptor subtype selectivity.^11^ Of particular note, the identification of allosteric modulators that exert pathway-specific effects on receptor signaling has given rise to a new subset known as biased allosteric modulators (BAMs).^7^ In addition to their allosteric activities, BAMs possess the ability to direct the responses to endogenous ligands towards either G protein- or arrestin-mediated pathways, a phenomenon referred to as biased signaling.^5,12^ Consequently, BAMs allow for the prevention of adverse side effects through their selection and maintenance of specific beneficial signaling pathways, thereby offering unprecedented opportunities for the development of safer, more targeted therapeutics.^8^ This potential has been highlighted by BAMs that target various GPCRs including the cannabinoid receptor type 1,^13,14^ CC chemokine receptors,^15–18^ the parathyroid hormone type 1 receptor (PTH1R),^19,20^ and the neurotensin receptor 1 (NTSR1),^7,21,22^ some of which have already advanced into clinical studies.^8^

To gain a comprehensive understanding of the pharmacology of BAMs and to explore strategies for their identification and development, intense studies have recently been conducted to elucidate the molecular mechanisms underlying their action. Very recent structures of PTH1R–G_s_ complex bound to the BAM PCO371,^19,20^ and NTSR1 in complex with G protein-coupled receptor kinase 2 (GRK2)^23^ or G_o_ protein,^24^ both in the presence of the BAM SBI-553, have provided structural insights into how BAMs bind to and modulate the conformation of these receptors. In a higher level of sophistication, these studies have unveiled how BAMs tune the interactions between GPCRs and specific signal transducers (G_s_, G_o_, GRK). Nonetheless, it still remains enigmatic how the transducer β-arrestin assembles on a GPCR in the presence of a BAM. This void leaves the molecular basis of BAMs’ capacity to tune arrestin-biased receptor signaling still only partially understood, thereby hindering the development of arrestin-biased therapeutic ligands for GPCRs aimed at circumventing G-protein signaling-associated side effects.

In this study, we unveil, for the first time, how GPCR interacts with arrestin in a BAM-bound state, by obtaining high-resolution cryo-EM structures (2.65–2.88 Å) of NTSR1 in complex with β-arrestin1 (βArr1) and the BAM SBI-553. As a class A GPCR, NTSR1 is activated by the endogenous peptide ligand neurotensin (NTS) and modulates dopaminergic neurotransmission and neuromodulation in the central nervous system.^25–27^ SBI-553 is a β-arrestin-biased allosteric activator of NTSR1 with the ability to bias NTS-occupied NTSR1 against G_q_ protein signaling and toward β-arrestin recruitment.^21,22^ This orally available, brain-penetrant lead compound has demonstrated potential in diminishing psychostimulant addiction behaviors, avoiding the typical side effects of hypotension, hypothermia, and motor impairment associated with conventional NTSR1 agonism.^21^ An important enabling factor in our study is the development of a robust chemical protein synthesis strategy to produce the full-length NTSR1 with defined hexa-phosphorylation at its carboxy-terminal tail (C-tail), which proves critical for obtaining a stable NTSR1–βArr1–SBI-533 complex. The well-resolved structures revealed an unprecedented “loop engagement” configuration, characterized by the intracellular loop 3 (ICL3) of NTSR1 docking into the cavity of the βArr1 central crest. Our structural investigations disclosed that the binding of SBI-553 to NTSR1 prompts substantial restructuring of the ICL3-TM6 and TM1-ICL1 receptor regions, culminating in an arrestin-selective conformation of NTSR1 that was not previously observed. Our work provides a structural framework for deepening our understanding of the nuanced mechanisms by which allosteric modulators can modulate GPCR–arrestin interactions and the subsequent biased activation of signaling pathways.

## Results

### Chemical synthesis of the phosphorylated NTSR1

**Fig. 1:**
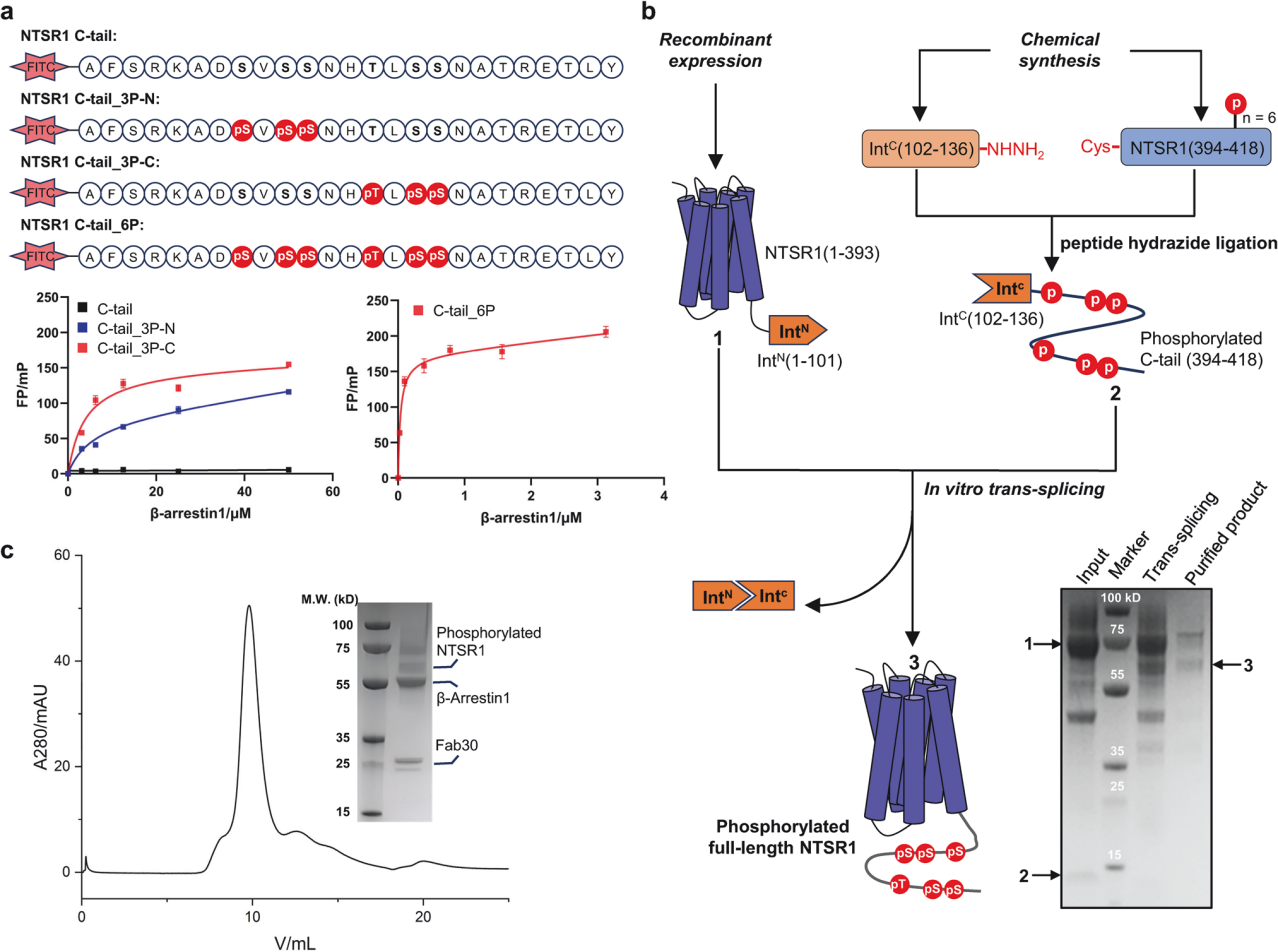
Construction of the NTSR1–βArr1 complex using a chemically synthesized phosphorylated NTSR1. **a** The binding affinities of phosphorylated peptides to βArr1 were evaluated by fluorescence polarization assay. Sequences of the chemically synthesized C-terminal peptides of NTSR1 bearing different phosphorylation patterns are shown. “pS” and “pT” denote phosphorylated serine and threonine residues, respectively, while FITC represents Fluorescein-5-isothiocyanate. **b** Schematic illustration of the semi-chemical synthesis of phosphorylated full-length NTSR1 using a *trans*-splitting approach. Phosphorylation sites are highlighted with red circles. **c** Size-exclusion chromatography and SDS-PAGE analysis of the NTSR1–βArr1–Fab30 complex construction.

Phosphorylation of NTSR1 in its C-tail by GRKs is crucial for the recruitment and activation of arrestins.^28,29^ Previous studies have revealed that the C-tail of NTSR1 bears multiple phosphorylation sites, among which S^401^-V^402^-S^403^-S^404^ and T^407^-L^408^-S^409^-S^410^ represent two plausible “*P*x*PP*” motifs (where P is a phosphorylation site).^30–32^ To assess the contribution of these two motifs to βarr1 engagement, we initially synthesized NTSR1 C-tail peptides bearing different phosphorylation patterns and validated their affinities to βArr1 by using the fluorescence polarization assay (Fig. 1a; Supplementary information, Fig. S1). The results indicated that peptides bearing phosphorylation at Ser^401^/Ser^403^/Ser^404^ or Thr^407^/Ser^409^/Ser^410^, exhibited comparable affinities for βArr1 in vitro, with dissociation constants (*K*_d_) of 5.11 μM and 3.77 μM, respectively (Fig. 1a). Notably, the peptide with hexa-phosphorylation at both motifs (Ser^401^/Ser^403^/Ser^404^ and Thr^407^/Ser^409^/Ser^410^) exhibited a remarkably enhanced affinity for βArr1, which was more than 100 times stronger (*K*_d_ = 35 nM) (Fig. 1a). This finding implies that the hexa-phosphorylated NTSR1 C-tail has the potential to recruit βArr1 to the receptor.

Traditionally, phosphorylated GPCRs were obtained either by co-expressing the receptors with GRKs^33–37^ or through in vitro phosphorylation by GRKs.^31,32,38^ However, obtaining homogeneous GPCR samples with a well-defined phosphorylation pattern poses a challenge when employing direct cellular expression or in vitro enzymatic methods. This difficulty can impede the formation of stable GPCR–arrestin complexes and hinder the high-resolution determination of how specific phosphorylation patterns orchestrate arrestin-mediated signaling pathways.^39,40^ To overcome this obstacle, GPCR-tail ligation strategies based on chemical protein synthesis have been developed to produce GPCRs with defined phosphorylation patterns, as exemplified by the phosphorylated receptors such as β1AR, β2AR and M2R.^41–45^ In this study, we set out to prepare full-length NTSR1 with a defined phosphorylation pattern at its C‑tail using a chemical protein synthesis method that combines peptide hydrazide ligation^44,46–48^ and intein-mediated protein *trans*-splicing ligation.^45,49^

We synthesized the phosphorylated C-tail segment of NTSR1 (residues 394–418, phosphorylated at Ser^401^/Ser^403^/Ser^404^ and Thr^407^/Ser^409^/Ser^410^), with the C-terminal segment of the CfaDnaE intein^50^ (residues 102–136) fused to its N-terminus. The 60-residue hexa-phosphorylated peptide was divided into two segments, each synthesized using routine microwave-assisted solid phase peptide synthesis method. Peptide hydrazide^46^ was employed to join the two peptide segments, yielding the desired intein-fused NTSR1 C-tail bearing hexa-phosphorylation (Fig. 1b). The transmembrane domain of human NTSR1 (residues 1–393), fused with the N-terminal segment of the CfaDnaE intein (residues 1–101) at its C-terminus, was over-expressed in HEK293F cells. The purified NTSR1 transmembrane domain and C-tail were then incubated to facilitate intein-mediated protein *trans*-splicing, yielding the full-length NTSR1 with a hexa-phosphorylation motif at its C-tail, ready for further structural analysis (Fig. 1b**)**.

### Assembly and structure determination of the NTSR1–βArr1–SBI-553 complex

For the assembly of the NTSR1–βArr1 complex, we utilized a constitutively active variant of human βArr1 that was truncated at residue 382 to eliminate autoinhibition. The complex formation process included the incorporation of the NTS_8–13_ peptide, comprising amino acids 8–13 of the NTS peptide sequence (RRPYIL). To enhance the stability of this complex, the well-characterized antibody fragment Fab30, which is specific for βArr1, along with PtdIns(4,5)P_2_, were integrated into the sample preparation process^32,35,36^ (Fig. 1c).

**Fig. 2:**
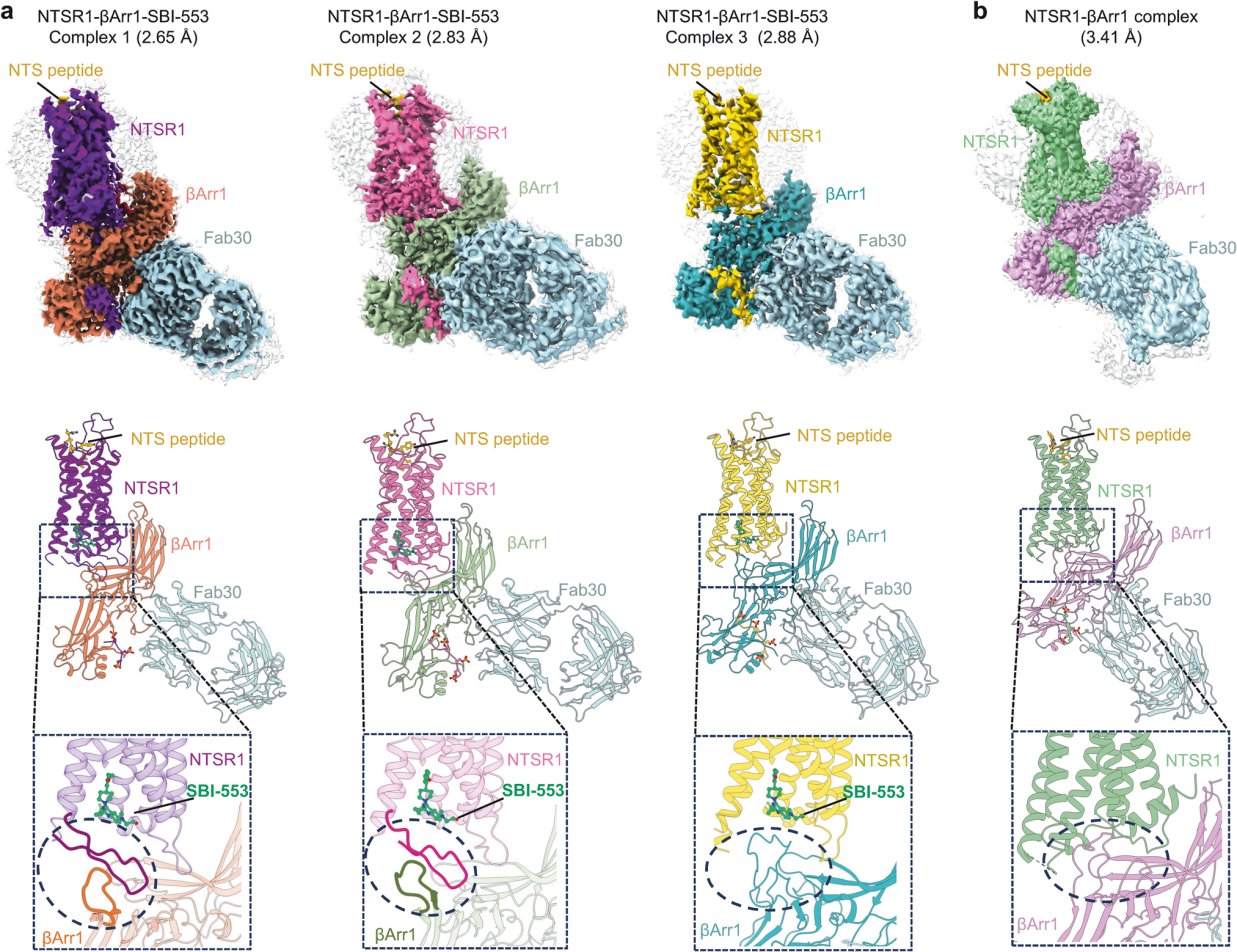
Cryo-EM structures of NTSR1–βArr1 complexes in SBI-553-bound or -unbound states. **a**, **b** Cryo-EM density maps and ribbon representations of three NTSR1–βArr1–SBI-553 complexes (**a**) and the NTSR1–βArr1 complex (**b**). The interfaces of βArr1 coupling to NTSR1 in each complex are indicated in magnified views.

The structures of the SBI-553-bound NTSR1–βArr1 complex and the NTSR1–βArr1 complex in the absence of SBI-553 were determined by cryo-EM. For the SBI-553-bound complex, a total of 17,359 images were collected, yielding ~5,000,000 particles after interactive 2-dimensional classifications. Further 3-dimensional reconstruction and refinement generated three distinct maps for the NTSR1–βArr1–SBI-553 complexes (referred to as complexes 1, 2, and 3), with global nominal resolutions of 2.65 Å, 2.83 Å and 2.88 Å, respectively (Fig. 2a; Supplementary information, Fig. S2 and Table S1). The 2.65 Å resolution map presented here represents the highest-resolution cryo-EM map for any GPCR–arrestin complex thus far.^30–37,42,43^ The maps for the three complexes were all sufficiently clear to place NTSR1, NTS_8–13_, βArr1, and the bound SBI-553. The peptide ligand NTS_8–13_ was observed within the top central pocket of the NTSR1 transmembrane domain, while SBI-553 was localized to the cytoplasmic pocket of the receptor (Fig. 2a). These maps also facilitated confident modeling of the majority of residues within the transmembrane helices and ICLs of NTSR1 (Supplementary information, Figs. S3 and S4). Additionally, the structure of NTSR1–βArr1 complex in the absence of SBI-553 was determined at a resolution of 3.41 Å, yielding a single 3-dimensional reconstruction (Fig. 2b).

The overall architectures of both the SBI-553-bound and -unbound NTSR1–βArr1 complexes display a high degree of similarity to those of the previously reported NTSR1–βArr1 complexes.^32^ This similarity is reflected in the comparable tilted angle between the longitudinal axis of βArr1 and NTSR1, as well as the slightly different perpendicular orientations of βArr1 relative to the GPCR bundle axis (Supplementary information, Figs. S5 and S6a, b). NTSR1–βArr1 complexes are stabilized by intermolecular interactions comprising two major interfaces: the intracellular region of NTSR1 transmembrane domain couples with the central crest of βArr1, and the C-tail of NTSR1 binds to the N-lobe of βArr1 (Fig. 2). Additionally, an interaction between the C-edge loop of βArr1 and detergent micelle is observed, consistent with the previously determined GPCR–arrestin complex structures.^32,42,43^ In complex 1 of the SBI-553-bound NTSR1–βArr1, a PtdIns(4,5)P_2_ molecule fits well into the density observed between the detergent micelle and the C-lobe of βArr1, likely contributing to the stabilization of the NTSR1–βArr1 complex as previously reported.^32^ For subsequent comparative structural analysis, we employed the previously reported NTSR1–βArr1 complex structure (PDB: 6UP7)^32^ as a reference.

### The distinct loop engagement configuration of the NTSR1–βArr1–SBI-553 complex

**Fig. 3:**
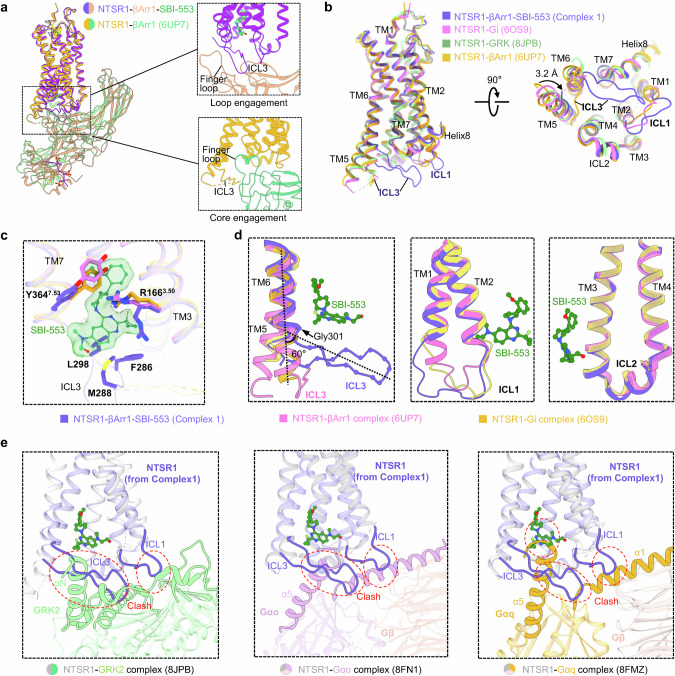
Analysis of the remodeled intracellular regions in the NTSR1–βArr1–SBI-553 complex 1. **a** Structural comparison between the NTSR1–βArr1–SBI-553 complex 1 and the NTSR1–βArr1 complex (PDB: 6UP7) reveals a distinctive “loop engagement” of βArr1 coupling to NTSR1 in the presence of SBI-553. **b** Comparative structural analysis of NTSR1 within the NTSR1–βArr1–SBI-553 complex 1 against those in the NTSR1–G_i_ complex (PDB: 6OS9), the NTSR1–GRK2 complex (PDB: 8JPB), and the NTSR1–βArr1 complex (PDB: 6UP7). **c** Magnified view of the Arg from the ERY motif and the Tyr from the NPxxY motif of NTSR1, derived from the NTSR1–βArr1–SBI-553 complex 1, NTSR1–G_i_ complex and NTSR1–βArr1 complex. **d** Comparative structural analysis focusing on the ICLs of NTSR1 from the NTSR1–βArr1–SBI-553 complex 1, NTSR1–G_i_ complex, and NTSR1–βArr1 complex. **e** Overlay of NTSR1 from the NTSR1–βArr1–SBI-553 complex 1 with those from NTSR1–GRK2, NTSR1–Gα_o_, and NTSR1–Gα_q_ complexes show that remodeled ICL1 and ICL3 in the “loop engagement” configuration would clash with GRK and G protein binding.

A close inspection of the high-resolution density maps for NTSR1–βArr1–SBI-553 complexes 1 and 2 disclosed a distinct configuration in which βArr1 couples to the intracellular side of NTSR1. In both complexes, a loop extending from the N-terminal of TM6, which corresponds to the ICL3 of NTSR1, was observed inserting into the cleft formed by central crest loops of βArr1, engaging βArr1 in a “hooking” manner (Fig. 3a). Interestingly, ICL3 was not resolved in most previously determined structures of GPCR–arrestin complexes due to its high flexibility. In previously reported structures, arrestin couples to GPCRs by inserting its bulk finger loop from the central crest into the intracellular transmembrane cavity of the receptor, which promotes the formation of stable receptor core–βArr1 interactions, known as the core engagement configuration.^32–35,42,43^ In complexes 1 and 2, the finger loop of βArr1 was positioned external to the transmembrane cavity of NTSR1, forming a pocket together with other loops from the central crest of βArr1 to engage the ICL3 of NTSR1. The unique configuration of NTSR1–βArr1 assembly in complexes 1 and 2, referred to as “loop engagement” hereafter, is observed in a GPCR–arrestin complex for the first time. We noted that superposition of complexes 1 and 2 reveals only minor in-plane rotations of βArr1 relative to the receptor, with no significant differences in the structure of the receptor or βArr1, or their interaction profiles (Supplementary information, Figs. S6c, d and S7). Given the structural similarity between complexes 1 and 2, we concentrated our subsequent analysis of the unique loop engagement configuration on complex 1 due to its superior resolution.

Structure comparison of the SBI-553-bound (complex 1) and -unbound NTSR1–βArr1 complexes (PDB: 6UP7)^32^ revealed extensive structural changes in the intracellular regions of NTSR1 between the two complexes, especially at the cytoplasmic sides of transmembrane helices and key motifs involved in NTSR1 activation and βArr1 coupling. Specifically, the cytoplasmic end of TM5 of NTSR1 from complex 1 shifted by 3.2 Å towards the receptor core compared to the SBI-553-unbound NTSR1–βArr1 complex, while other TMs showed only minor shifts (Fig. 3b). In the intracellular cavity, the SBI-553 binding disrupted the contact between R166^3.50^ from the “ERY” motif and Y364^7.53^ from the “NPxxY” motif^51^ (Fig. 3c). More significantly, the cytoplasmic end of TM6 (residues R294^6.26^–H300^6.32^) adopted an extended loop structure, deviating from the typical continuous helical conformation. The extended loop region bent towards the central cavity at G301^6.33^, forming an angle of ~60° with respect to the TM6 axis (Fig. 3d). At the N-terminus of this extended region, a loop composed of 10 residues forms a hairpin-like structure, corresponding to the C-terminal part of ICL3. This ICL3 conformation is distinct from that observed in the SBI-553-unbound NTSR1–βArr1 complex, where the C-terminal end of ICL3 makes a sharp turn at the end of TM6 and extends posterior to TM5 (Fig. 3d). In addition to the changes in TM6 and ICL3, the ICL1 of NTSR1 was also remodeled in the SBI-553-bound complex compared to the SBI-553-unbound NTSR1–βArr1 complex. The cytoplasmic end of TM1 (residues A89–S93) also adopted an extended structure, resulting in a more extended ICL1 that swings away from the receptor core (Fig. 3d).

When compared with previously reported SBI-553-bound NTSR1 structures,^23,24^ the newly determined structures presented here reveal more comprehensive contacts between SBI-553 and the remodeled NTSR1 intracellular region. Consistent with that observed in the structures of SBI-553-bound NTSR1–Gα_o_ and NTSR1–GRK complexes, SBI-553 forms predominately hydrophobic interactions with residues from TM2, TM3, TM5, TM6, TM7 and Helix 8 in the NTSR1–βArr1 complex (Supplementary information, Fig. S8a). Beyond these transmembrane segments, residues M298, F286, and L298, located in the extended ICL3 region, also interact with SBI-553 (Fig. 3c**)**, which were not observed in the SBI-553-bound NTSR1–Gα_o_ or NTSR1–GRK complexes. Specifically, the quinazoline group of SBI-553 is engaged in hydrophobic interactions with the side chains of M298, F286, and L298. Alanine substitution of these residues resulted in the decreased affinities of SBI-553 for binding to NTSR1, which were reflected by higher EC_50_ values of SBI-553 acting on the receptor (Supplementary information, Fig. S8b).

The distinct conformation of the intracellular region of NTSR1 observed in the loop engagement configuration is presumably an arrestin-selective conformation of the receptor. When aligned with the previously reported structures of NTSR1–GRK, NTSR1–Gα_q_, and NTSR1–Gα_o_ complexes, the NTSR1 in our structure reported here, which has undergone remodeling in the ICL3-TM6 and TM1-ICL1 regions, clearly hinders the binding of GRK or G proteins (Fig. 3e). To be specific, the extended ICL3 clashes with the αN helix of GRK2, as well as with the αN helices of Gα_q_ or Gα_o_ proteins. Moreover, the remodeled ICL1 also creates steric conflicts with GRK, Gα_q_, or Gα_o_ proteins. These steric conflicts demonstrate an arrestin-selective conformation of NTSR1, which is only observed in our structure, rather than the previously reported NTSR1–GRK and NTSR1–Gα_o_ complexes. These conformational changes may serve as a facilitating factor, enabling SBI-553 to direct NTSR1 signaling towards arrestin.

### Structural basis of the loop engagement configuration

**Fig. 4:**
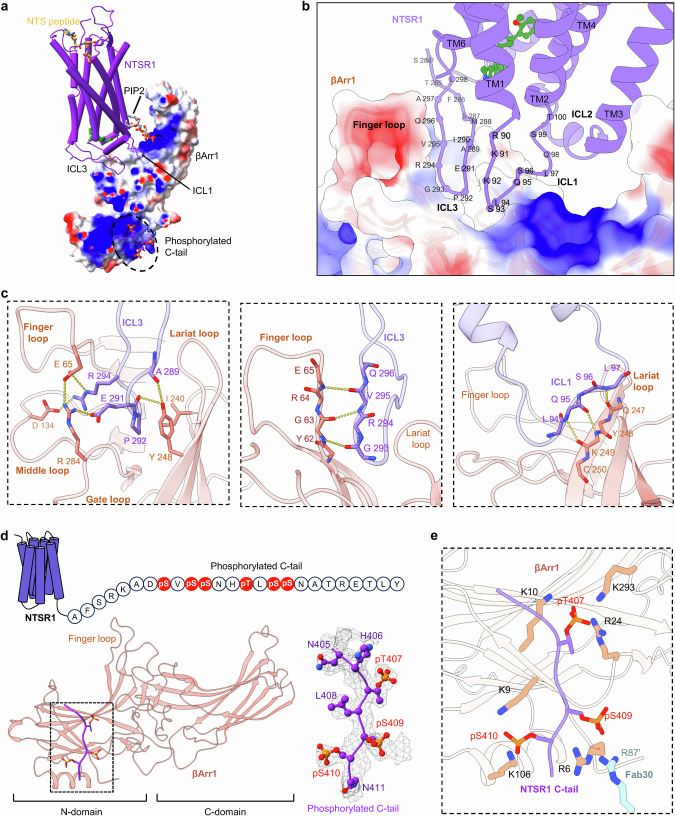
Interactions between NTSR1 and βArr1 in the loop engagement configuration. **a** Overall structure of the NTSR1–βArr1–SBI-553 complex 1. **b** The intracellular interface between NTSR1 and βArr1 in the NTSR1–βArr1–SBI-553 complex 1. **c** Detailed interactions at the major interface between the NTSR1 ICLs (ICL1 and ICL3) and βArr1. NTSR1 is shown in violet and βArr1 is shown in coral. **d**, **e** βArr1 structure derived from the NTSR1–βArr1–SBI-553 complex 1 illustrates the interactions between the phosphorylated C-tail and βArr1 (**d**). Interactions between the phosphorylated residues in NTSR1 C-tail and the positive charged residues in βArr1 and Fab30 are indicated (**e**).

In complex 1, the clear density map reveals detailed intermolecular interactions between NTSR1 and βArr1 at the residue-specific level within the context of the loop engagement configuration. The interaction between NTSR1 and βArr1 involves two primary interfaces: a major interface consisting of the ICL3 loop of NTSR1, which inserts into the open cavity of the central crest of βArr1, and a minor interface consisting of the ICL1 of NTSR1, which interacts with the lariat loop of βArr1 (Fig. 4a, b). The total buried surface area between βArr1 and NTSR1 in complex 1 is ~1049 Å^2^ (calculated by using PDBePISA), with 8 hydrogen bonds and 2 salt-bridges observed.

At the major interface, the hairpin-like ICL3 loop, which made a turn at P292/G293 site, penetrates deeply into the cavity in the crest of βArr1 formed by the finger, middle, gate and lariat loops (Fig. 4c; Supplementary information, Fig. S9a). Residues E291 and R294 in ICL3 of NTSR1 make contacts with E65, D134 and R284 in βArr1 via an electrostatic interaction network. Additionally, P292 from ICL3 is tightly packed against the side chains of Y248 and L240 from βArr1. The main-chain carbonyl groups of A289 and P292 also form direct hydrogen bonds with the side chain of Y248 (Fig. 4c). Consistent with their roles in the NTSR1–βArr1 interactions, mutations of these key interface residues in NTSR1 and βArr1 mentioned above resulted in reductions in SBI-553-mediated βArr1-biased signaling of NTSR1 (Supplementary information, Fig. S10). Moreover, residues G293, R294, V295, and Q296 from ICL3 are closely packed against Y62, G63, R64, and E65 from the finger loop of βArr1, forming multiple hydrogen bonds via their main chains (Fig. 4c). At the ICL1 minor interface, residues from L94 to L97 from ICL1 are arranged in an antiparallel-like manner against residues Q247 to C250 from the lariat loop of βArr1, forming multiple hydrogen bonds via their main chains (Fig. 4c; Supplementary information, Fig. S9b). Notably, the analysis of βArr1 recruitment revealed that alanine substitutions of F286, M288, and L298 also resulted in a diminished effect of SBI-553 in promoting βArr1 recruitment (Supplementary information, Fig. S10). Although there is no evidence that these residues directly interact with βArr1, their contribution to SBI-553 binding to NTSR1 is likely the cause of this observation.

### Binding of the phosphorylated NTSR1 C-tail to βArr1

In addition to the intracellular region of the NTSR1 transmembrane domain coupling to the central crest of βArr1, the C-tail of NTSR1 was also observed to bind the N-lobe of βArr1. In all three NTSR1–βArr1 complex structures solved here, we observed densities corresponding to part of the C-tail of NTSR1 docking into the N-lobe of βArr1, which displaces the C-terminus of βArr1 by binding to a positively charged crevice. Three phosphate groups on T407/S409/S410 were unambiguously assigned (Fig. 4d). The organized *P*-x-*P*-*P* pattern was engaged in charge complementarity-based interactions with selected Lys and Arg residues in the N-lobe groove of βArr1, including R6, K9, K10, R24, K106 and K293 (Fig. 4d). Specifically, the phosphorylated T409 (pT407) forms charge–charge interactions with K10, R24 and K293. pS409 engages via both polar and charge–charge interactions with R6 in the βArr1 and R87 in the Fab30. pS410 makes contacts with K9 and K106 in βArr1 (Fig. 4d). These observations establish the key contribution of the T-L-S-S motif in the C-tail in driving βArr1 recruitment and activation.

The binding model of this single “*P*x*PP*” motif displays high similarity to that observed in the crystal structure of βArr1 in complex with a fully phosphorylated C-tail peptide derived from the human V2 vasopressin receptor (V2R). The orientation and coordination of the phosphate groups within the three pS/pT residues of NTSR1 C-tail are almost identical to those of the V2R C-tail peptide (Supplementary information, Fig. S11a). However, while NTSR1 possesses two phosphorylated motifs, the N-terminal “*P*x*PP*” motif in the phosphorylated NTSR1 C-tail (S401-N402-S403-S404), which has been proven important in peptide binding to βArr1, was not observed in our structure. This contrasts with the binding mode of the fully phosphorylated V2R C-tail peptide to βArr1, where the N-terminal phosphorylated motif also engages with βArr1. This indicates that the binding mode of multiply phosphorylated GPCR C-tails to arrestins may differ in the context of truncated peptides and intact receptors.

The βArr1 in complex 1 presented here adopts a structure replete with the conformational signatures of activated arrestin, where its finger, gate, and middle loops from the central crest, which is essential for receptor coupling, align in active-state conformations. Nonetheless, upon superimposing the structures of βArr1 within NTSR1–βArr1 complexes, in the absence or presence of a BAM, it becomes evident that the finger loops of βArr1 assume distinct conformations (Supplementary information, Fig. S11b). These findings indicate that the flexible finger loops of active βArr1 are capable of adopting a range of conformations, which likely facilitates βArr1’s ability to engage with diverse receptor cavities.

### Conformational plasticity of βArr1 coupling to NTSR1

**Fig. 5:**
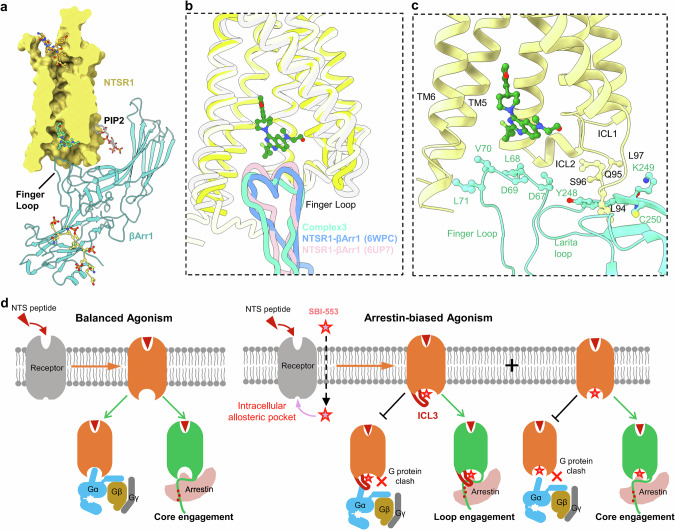
Interactions between NTSR1 and βArr1 in a core engagement configuration in the presence of SBI-553. **a** Overall view of the core engagement configuration of NTSR1–βArr1–SBI-553 complex 3. **b** Superposition of NTSR1 from the complex 3, NTSR1–βArr1 complex (PDB: 6PWC) and the NTSR1–arrestin complex (PDB: 6UP7) shows that the finger loop of βArr1 is compatible with the location of SBI-553. **c** The intracellular interface between NTSR1 and βArr1 in the NTSR1–βArr1–SBI-553 complex 3. **d** Schematic illustration of the proposed working model of arrestin-biased agonism of NTSR1 potentiated by SBI-553.

In contrast to the unique loop configuration observed in the complexes 1 and 2, the complex 3 of NTSR1–βArr1–SBI-553 was found to adopt the conventional core engagement configuration, where βArr1 engages NTSR1 extensively at the intracellular transmembrane cavity and ICL regions via its finger loop from the central crest (Fig. 5a, b). TM6 of NTSR1 maintains a typical continuous helical structure, and the extended loop associated with ICL3 is not observed. A comparison between complex 3 and the SBI-553-unbound NTSR1–βArr1 complex (PDB: 6UP7)^32^ reveals similar NTSR1 structures (r.m.s.d. = 0.785 Å for all the Cα atoms of the receptor) but shows a swing of βArr1, suggesting that complex 3 adopts the canonical core engagement found in previously reported GPCR–arrestin complexes (Supplementary information, Fig. S6a).

In complex 3, the SBI-553 molecule is positioned at the NTSR1–βArr1 interface in a pose identical to that observed in complex 1 and complex 2 (Supplementary information, Fig. S7). The structure of complex 3 clearly reveals that SBI-553 forms interactions with both NTSR1 and βArr1. Specifically, the pendant phenyl ring along with its methoxy substituent in SBI-553 is engaged by the hydrophobic intracellular transmembrane cavity of NTSR1. The quinazoline group points outside the cavity and has contacts with L68 and V79 in the finger loop of βArr1 (Fig. 5c).

When compared with the SBI-553-unbound NTSR1–βArr1 complexes, the finger loop of βArr1 in complex 3 is pushed down, probably due to the location of SBI-553. Nevertheless, SBI-553 does not impede the interaction between βArr1 and NTSR1. The finger loop of βArr1 remains engaged by the lower part of the intracellular transmembrane cavity of NTSR1. In addition, the remodeled ICL1 of NTSR1 forms hydrogen bonds through main-chain interaction with the lariat loop of βArr1 (Fig. 5c). The total interface area between βArr1 and NTSR1 in complex 3 is ~725 Å^2^, significantly smaller than the NTSR1–βArr1 interface area in complex 1 (1049 Å²), suggesting relatively weaker NTSR1–βArr1 interactions. Collectively, the binding of SBI-553 is compatible with arrestin binding to NTSR1, consistent with its arrestin-biased signaling property. The three structures of NTSR1–βArr1–SBI-553 complex determined here reveal the coexistence of core engagement and loop engagement configurations of βArr1 coupling to NTSR1 in the presence of SBI-553, indicating a high degree of conformational plasticity and diverse interface contacts between NTSR1 and βArr1, potentially enabling SBI-553 to orchestrate arrestin-biased signaling of NTSR1.

## Discussion

In this study, we present the structural characterization of the NTSR1–βArr1 complex in the presence of SBI-553, an arrestin-biased intracellular allosteric modulator of NTSR1. We disclose the first structure of a GPCR–arrestin complex bound with a BAM and present the highest-resolution density map among the currently available GPCR–arrestin complexes. A key enabling factor in our research is the development of a robust chemical protein synthesis strategy for generating full-length NTSR1 with defined hexa-phosphorylation at its C-tail. This strategy has proven to be of utmost importance in obtaining a stable NTSR1–βArr1 complex.

The well-resolved structures offer precise and unambiguous molecular details of the interactions between NTSR1 and βArr1 in the presence of SBI-553, along with the specific binding mode of SBI-553 in the βArr1-coupled state. It is particularly worthy of highlighting that our structures have uncovered a unique “loop engagement” configuration in the coupling of βArr1 to NTSR1 when SBI-553 is present. To the best of our knowledge, this “loop engagement” configuration represents a previously unobserved mode of arrestin coupling to GPCRs, differentiating it from the well-known “core engagement” and “tail engagement” modes. This configuration is primarily characterized by the ICL3 of NTSR1 docking into the cavity of the βArr1 central crest, rather than the receptor’s intracellular transmembrane cavity engaging the finger loop of βArr1, as seen in the canonical “core engagement” configuration. We also determined the structure of the NTSR1–βArr1 complex in the absence of SBI-553 utilizing the chemically synthesized, phosphorylated NTSR1. Intriguingly, only the typical “core engagement” configuration was observed upon βArr1 binding to NTSR1, with no sign of the “loop engagement”. These findings collectively imply that the uniquely observed “loop engagement” configuration of βArr1 does not represent a naturally occurring phenomenon in the absence of SBI-553, but is more likely attributed to, and requires the binding of SBI-553. This report highlights the novel functionality of SBI-553 in its ability to stabilize a distinct and potentially artificially induced conformation of β-arrestin coupling. At present, it remains elusive whether this conformation is a general phenomenon of arrestin-BAMs acting on GPCRs or is specific to the action of SBI-553 on NTSR1. To shed light on this, further investigations are needed to elucidate the structural mechanism of diverse BAMs in modulating the coupling between arrestin and GPCRs.

G protein- or arrestin-BAMs have become a recent focus of GPCR drug discovery studies due to their potential to selectively modulate the interaction between GPCRs and specific signal transducers. SBI-553, acting as an arrestin-BAM of NTSR1, has demonstrated its potential to produce a biased stimulation of the receptor toward β-arrestin and away from Gα_q_ protein.^21^ The recently reported cryo-EM structures of NTSR1–GRK2 and NTSR1–Gα_o_ complexes, both bound with SBI-553, have revealed that SBI-553 occupies the intracellular transmembrane cavity of NTSR1.^23,24^ Structural analysis suggested that the binding mode of SBI-553 is compatible with GRK2/Gα_o_ but conflicts with Gα_q_ binding. Here, our structure provides structural evidence indicating that the binding of SBI-553 is also compatible with arrestin binding, within an unexpected “loop engagement” configuration. Interestingly, an SBI-553-bound “core engagement” configuration is also observed. The specific binding modes of SBI-553 in NTSR1 could serve as the basis of the arrestin signaling selectivity of SBI-553. Moreover, the observed concurrent increases in orthosteric ligand affinity and preferential β-arrestin coupling have suggested that SBI-553 can induce high-affinity, β-arrestin-selective receptor conformations.^7,21^ In the distinctive loop engagement configuration observed in our studies, the ICL3-TM6 and TM1-ICL1 regions of NTSR1 undergo significant conformational changes when compared with the typical core engagement configuration. This could facilitate the recruitment of βArr1 by forming extensive interactions with the loops from the central crest of βArr1. We hypothesize that the remodeled ICL3-TM6 and TM1-ICL1 regions in the loop engagement configuration represent the proposed arrestin-selective conformation. This arrestin-selective conformation provides more extensive contacts for βArr1 coupling to NTSR1, involving ICL1 and ICL3 from the receptor and the central crest loops form βArr1. This might consequently lead to the enhanced recruitment of βArr1 to the receptor in the presence of SBI-553. SBI-553 thus has demonstrated its ability to tune the recruitment of arrestin to NTSR1 by utilizing the structure plasticity of the receptor. Collectively, the structures that we reported here, combined with the structures of NTSR1 in complex with GRK2 or Gα_o_, both in the SBI-553-bound state, provide a comprehensive view of the molecular basis that enables the intracellular biased modulator SBI-553 to facilitate arrestin-biased signaling in NTSR1.

In summary, our findings bridge the critical knowledge gap in the regulation of NTSR1–arrestin interactions and biased signaling by SBI-553. By integrating our results with existing structural data of NTSR1 in complex with G protein or GRK, either in the presence or absence of SBI-553, as well as the functional profiles of SBI-553 observed in cell lines and animal models, our study elevates SBI-553 to a select group of the most well-characterized allosteric xenobiotics known to elicit biased receptor signaling. Our work is valuable in enhancing our understanding of GPCR biology and have significant implications for the design and development of biased chemical compounds for GPCR-targeted therapeutics. Despite these advantages, there exist other allosteric modulators and BAMs with mechanisms distinct from that of SBI-553, such as variations in the conservation of their binding sites across different receptors and differences in the potential mechanisms by which they modulate receptor signaling. Given their diversity and complexity, a comprehensive understanding of the action mechanisms of BAMs requires subsequent investigations into the modulation of GPCR structure and signaling by various BAMs, thereby providing a more complete picture of the field.

## Materials and methods

### Peptide chemical synthesis

For the synthesis of NTSR1 C-tail (residues 394–418) bearing hexa-phosphorylation, the 2-chlorotrityl chloride was swelled with dichloromethane/dimethylformamide (DCM/DMF; 1/1, v/v) for 30 min. The first tyrosine (4.0 equivalents (eq)) was resolved in DMF and DIEA (8.0 eq) and reacted overnight. The Fmoc group was removed with piperidine (20% in DMF, 5 min +10 min). Again, the resin was washed with DMF (3 times), DCM (3 times) and DMF (3 times). The second leucine (4.0 eq) was pre-activated with *O*-(6-Chloro-1-hydrocibenzotriazol-1-yl)-1,1,3,3-tetramethyluronium hexafluorophosphate (HCTU; 4.0 eq) and diisopropylethylamine (DIEA; 8.0 eq) in DMF for 0.5–1 min. Then, the mixture was added to the resin for coupling. After 30 min, the resin was washed with DMF (3 times), DCM (3 times) and DMF (3 times). The following amino acid residues (3–8) were coupled to the resin with the same procedure. Starting from the ninth amino acid, we used a novel approach to synthesize peptides containing phosphorylation. The Fmoc group was removed with a new condition (2% 1-Hydroxybenzotriazole (HOBt), 2% hexamethyleneinine, 25% *N*-methylpyrrolidine, in DMSO-*N*-Methyl-2-pyrrolidone (1:1), 5 min + 5 min). The phosphorylated serine (4.0 eq) was pre-activated with 2-(7-Azabenzotriazol-1-yl)-N,N,N',N'-tetramethyl uronium hexafluorophosphate (HATU; 4.0 eq), 1-Hydroxy-7-azabenzotriazole (HOAt; 4 eq) and DIEA (8.0 eq) in DMF for 0.5–1 min. Then, the mixture was added to the resin for coupling at 50 °C for 15 min twice. The following all amino acid residues were coupled to the resin with the same procedure. After the solid phase amino acid assembly, the completed peptide was cleaved from the resin with a mixture of trifluoroacetic acid (TFA)/water/phenol/Triisopropylsilylacetylene (TIPS) (88/5/5/2, v/v/v/v). After 2 h, the TFA-containing solution was collected and concentrated by blowing with N2. The crude peptide was obtained by precipitation with cold ether and centrifugation. The residue was dissolved in water/acetonitrile (1:1, 0.1% TFA), purified by HPLC and analyzed by high-resolution ESI mass spectra.

For the synthesis of CfaDnaE-Int^C^ hydrazide peptide, the 2-chlorotrityl chloride was swelled with DCM/DMF (1/1, v/v) for 30 min. The hydrazine hydrate was added to the peptide synthesis tube until the resin was submerged and reacted for 2 h; the above procedure was repeated. The first Ala (4.0 eq) was pre-activated with HCTU (4.0 eq) and DIEA (8.0 eq) in DMF for 0.5–1 min. Then, the mixture was added to the resin for coupling. After 30 min, the resin was washed with DMF (3 times), DCM (3 times) and DMF (3 times). The Fmoc group was removed with piperidine (20% in DMF, 5 min +10 min). Again, the resin was washed with DMF (3 times), DCM (3 times) and DMF (3 times). The following amino acid residues were coupled to the resin with the same procedure. After the solid phase amino acid assembly, the completed peptide was cleaved from the resin with a mixture of TFA/water/phenol/TIPS (88/5/5/2, v/v/v/v). After 2 h, the TFA-containing solution was collected, and concentrated by blowing with N_2_. The crude peptide was obtained by precipitation with cold ether and centrifugation. The residue was dissolved in water/acetonitrile (1:1, 0.1% TFA), purified by HPLC and analyzed by high-resolution ESI mass spectra.

For the ligation of Int^C^ hydrazide peptide and phosphorylated NTSR C-tail, the hydrazide peptide (1.0 eq, final concentration 1–3 mM) was dissolved in acidic ligation buffer (the aqueous buffer containing 6 M guanidine hydrochloride (Gn·HCl) and 200 mM NaH_2_PO_4_, pH = 3.0). Then the above solution was cooled to –11 °C to –13 °C by an ice-salt bath. Then, the peptide solution was treated with 200 mM NaNO_2_ solution (6.5 eq, dissolved in the acidic ligation buffer) and subsequently stirred for 25–30 min (under –11 °C to –13 °C). Then, a solution of 65 eq of 200 mM methylphenylacetic acid (MPAA; dissolved in the aqueous buffer containing 6 M Gn·HCl and 200 mM Na_2_HPO_4_, pH = 7.0) was added. The reaction mixture was then taken out from the ice-salt bath and stirred for 3 min at room temperature. Then, the phosphorylated NTSR1 C-tail peptide (1.0–1.2 eq) was added. Then, the pH value of the reaction mixture was slowly adjusted to 6.5–6.9 with 2 M NaOH (aqueous solution). Then, the reaction was stirred at room temperature. Analytic RP-HPLC and ESI-MS were used to monitor the reaction process. After the completion of the reaction, the mixtures were treated with 200 mM Tris(2-chloroethyl) phosphate (TCEP; equal volumes to the reaction system). Finally, the ligation product was purified by semi-preparative RP-HPLC and lyophilized. Especially, the auxiliary-mediated ligation of peptide hydrazide between peptide 3 and peptide 6 was conducted using the protocols described above.

### Constructs

The truncated form of human NTSR1 (residues 1–393) fused with CfaDnaE^N101^ at its C-terminus was codon-optimized for expression in HEK293 cells and cloned into a modified pCDNA3.1 vector, which contains an N-terminal hemagglutinin (HA) signal peptide followed by a b562RIL epitope and HRV 3C site before the receptor. Human βArr1 was truncated at residue 382 to eliminate autoinhibition and an 8× His tag was added at its N-terminus. The truncated form was cloned into a pET28a vector for overexpression in *Escherichia coli* BL21(DE3) cells. For the antibody fragment Fab30, both the light chain and heavy chain were modified with a GP64 secretion signal peptide at the N-terminus. A C-terminal 8× His tag was added to the heavy chain. The genes for modified light chain and heavy chain were codon-optimized and cloned into pFastbac-dual vector for expression in *Spodoptera frugiperda* (*Sf*9) insect cells.

### Protein expression and purification

The modified human NTSR1 was expressed in HEK293F cells. Cells were grown to a density of 2–3 × 10^6^ cells per mL of culture and transfected with the plasmid. After 48 h, cells were harvested by centrifugation at 3700× *g* for 10 min. Cell pellets were collected and lysed in lysis buffer (20 mM HEPES, pH 7.4, 500 mM NaCl) supplemented with Protease Inhibitor Cocktail (EDTA-Free) and 100 μM TCEP, followed by Dounce homogenization. The lysate was ultracentrifuged at 200,000× *g* at 4 °C for 30 min. The raw membrane was resuspended by Dounce homogenization in the lysis buffer and solubilized using 1.0% (w/v) lauryl maltose neopentyl glycol (LMNG; Anatrace), 0.1% (w/v) cholesterol hemi succinate (CHS; Sigma) for 2 h at 4 °C. The supernatant was collected by ultracentrifugation at 200,000× *g* for 45 min and then incubated with G1 anti-Flag affinity resin (Genscript) for 1 h at 4 °C. After incubation, the resin was loaded into a plastic gravity flow column and washed with 30 column volumes of washing buffer (20 mM HEPES, pH 7.4, 500 mM NaCl, 0.005% (w/v) LMNG, 0.001% (w/v) CHS, 5 μM NTS_8–13_, 100 μM TCEP, 2 mM CaCl_2_). The protein was eluted with 10 column volumes of elution buffer (20 mM HEPES, pH 7.4, 300 mM NaCl, 0.001% (w/v) LMNG, 0.0002% (w/v) CHS, 1 μM NTS_8–13_, 100 μM TCEP, 2 mM CaCl_2_, 10 μM SBI-553 (TargetMol) and 0.2 mg/mL Flag peptide). The eluted NTSR1 protein was concentrated using a 50 kDa molecular weight cut-off Centrifugal Filter and then loaded into a Superose6 Increase 10/300 GL column (GE Healthcare) equilibrated in running buffer containing 20 mM HEPES, pH 7.4, 300 mM NaCl, 0.001% (w/v) LMNG, 0.0002% (w/v) CHS, 1 μM NTS_8–13_, 100 μM TCEP, 2 mM CaCl_2_, 5 μM SBI-553. The fractions for the monomeric NTSR1 were collected and concentrated for intein *trans*-splicing ligation.

The cells expressing βArr1 were grown in LB medium supplemented with 50 μg/mL kanamycin at 37 °C for 3 h and then cultured at 16 °C overnight after addition of 200 μM IPTG. The cells were harvested by centrifugation at 3700× *g* for 20 min and lysed in 20 mM HEPES (pH 7.4), 150 mM NaCl, 5 mM β-mercaptoethanol by sonication. The supernatant was collected by centrifugation at 12,000× *g* for 30 min and loaded to Ni-NTA affinity chromatography. The protein bound to the Ni-NTA resin was washed by buffer A (20 mM HEPES, 500 mM NaCl, 5 mM β-mercaptoethanol), buffer B (20 mM HEPES, 1 M NaCl, 5 mM β-mercaptoethanol), buffer C (20 mM HEPES, 500 mM NaCl, 5 mM β-ME, 40 mM imidazole) in turn and further eluted with 5 column volumes of elute buffer containing 20 mM HEPES, 200 mM NaCl, 5 mM β-mercaptoethanol, 300 mM imidazole. The protein sample was concentrated and purified on a Superdex200 Increase 10/300 column (GE Healthcare) using running buffer (20 mM HEPES, pH 7.4, 200 mM NaCl and 5 mM β-mercaptoethanol). The peak fractions were collected and concentrated, flash-frozen in liquid nitrogen and store at –80 °C until use.

Fab30 was expressed in *Sf*9 cells using a Bac-to-Bac-derived Baculovirus expression system. Cells were infected at a density of 2.8 × 10^6^ cells per mL of culture and harvested 60 h post infection. Cells were pelleted by centrifugation and the supernatant was transferred to a large plastic container and incubated with the Ni-NTA agarose for 1 h on ice. The resin was washed with 20 mM HEPES (pH 7.4), 500 mM NaCl, 20 mM imidazole and then with 20 mM HEPES (pH 7.4), 100 mM NaCl, 40 mM imidazole. Fab30 was eluted with 20 mM HEPES (pH 7.4), 100 mM NaCl, 400 mM imidazole and concentrated, further subjected to polishing by size-exclusion chromatography on a Superdex200 10/300 column (GE Healthcare) equilibrated with 20 mM HEPES (pH 7.4), 100 mM NaCl, 5% (v/v) glycerol. Peak fractions were pooled and concentrated to 2 mg/mL, flash-frozen in liquid nitrogen and store at –80 °C until use.

### Intein *trans*-splicing ligation

The ligation was prepared as previously described.^45,52^ In brief, lyophilized DnaE-fused phosphorylated C-tail peptide of NTSR1 was dissolved directly in NTSR1 concentrate samples at a molar ratio of NTSR1:phosphorylated peptide = 1:5, and supplemented with TCEP and EDTA to final concentrations of 500 μM and 1 mM, respectively. The reaction components were thoroughly mixed and incubated overnight at 25 °C. After incubation, the phosphorylated NTSR1 was purified by reverse G1 anti-Flag affinity resin (Genscript) to remove unligated NTSR1, then concentrated for subsequent complex assembly.

### Fluorescence polarization (FP) assays

All the FP assays were performed in buffer consisting of 20 mM HEPES, pH 7.4, 100 mM NaCl and 0.01% (w/v) DDM on a 96-well flat-bottom OptiPlate black plate using an Infinite 200 PRO microplate reader. For assays of the binding of peptides NTSR1 C-tail, NTSR1 C-tail_3P-N and NTSR1 C-tail_3P-C to arrestin, a 6-point dilution series of arrestin (0, 3.125, 6.25, 12.5, 25, 50 μM) was prepared in EP tubes. For assays of the binding of peptides NTSR1 C-tail_6P to arrestin, a 7-point dilution series of arrestin (0, 0.025, 0.10, 0.40, 0.80, 1.60, 3.20 μM) was prepared. The peptides were added at a final concentration of 4 nM to each tube. Samples were incubated at room temperature for 30 min and transferred to a 96-well plate at 50 μL/well. The reading was performed by default settings of the Infinite 200 PRO microplate reader, and excitation‑/emission-wavelengths of 485 nm/535 nm were used. The obtained data were fitted using the “One Site–Total” (for saturation binding) nonlinear regression methods with Graphpad Prism software:

$${{{\rm{Y}}}}={{{\rm{B}}}}\max \times {{{\rm{X}}}}/({K}_{{{{\rm{d}}}}}+{{{\rm{X}}}})+{{{\rm{NS}}}}\times {{{\rm{X}}}}+{{{\rm{Background}}}}$$

where Y stands for anisotropy, Bmax is the maximum anisotropy of the protein–ligand complex in the same units as Y, X is the concentration of the added ligand, *K*_d_ is the equilibrium dissociation constant in the same units as X, NS is the slope of the nonlinear regression in Y units divided by X units, Background is the measured anisotropy with no added ligands. All FP assays were performed in triplicate. All FP assays were performed in triplicate.

### Complex construction, cryo-EM grid preparation and data collection

For complex construction, the phosphorylated NTSR1 was first mixed in an equimolar ratio with βArr1 and diC8-PtdIns(4,5)P_2_ at a concentration of ~5 μM and supplemented with NTS_8–13_ to a final concentration of 10 μM. The mixture was incubated at 25 °C for 30 min. Then Fab30 was added at a molar ratio of Fab30:βArr1 = 1.5:1 and incubated at 25 °C for 90 min. The NTSR1–βArr1–Fab30 complex was concentrated with a 100 kDa molecular weight cut-off Centrifugal Filter and then loaded into a Superose6 10/300 GL column (GE Healthcare) equilibrated in the buffer containing 20 mM HEPES (pH 7.4), 100 mM NaCl, 0.001% (w/v) LMNG, 0.0002% (w/v) CHS, 1 μM NTS_8–13_, 100 μM TCEP. The peak fractions containing the complex were combined and concentrated to 1 mg/mL for cryo-EM sample preparation.

The cryo-EM samples were prepared by applying an aliquot of 3 μL protein sample of NTSR1–βArr1–Fab30 complex to a glow-discharged holey grid (ANTcryoTM R1.2/1.3, Au 300 mesh) and flash frozen in liquid ethane using a Mark IV Vitrobot (Thermo Fisher Scientific) with a blot time of 4 s and a blot force of 1 at 4 °C and 100% humidity. Dataset of NTSR1–βArr1 complex was collected by a Titan Krios G4 at 300 KV accelerating voltage, equipped with a Gatan K3 direct electron detector at Advanced Center for Electron Microscopy at Shanghai Institute of Materia Medica (SIMM), Chinese Academy of Sciences. Micrographs were recorded with pixel size of 0.824 Å. In total, 17,359 movies were obtained at a dose of 50 electron per Å^2^ for 36 frames. The defocus range of this dataset was –0.8 μm to –2 μm.

### Cryo-EM data processing and three-dimensional reconstruction

For the NTSR1–βArr1 complex, movies were aligned with relion-3.1.^53^ Initial contrast transfer function fitting was performed with CTFFIND4.1^54^ from Cryosparc. Automated particle selection yielded many particle projections from three automated tools, producing 17,456,259 particles for further processing. The projection was subjected to reference-free 2-dimensional classification to discard poorly defined particles, producing 5,006,335 particle projections. With the initial model from Ab-Initio Reconstruction, multiply heterogeneous refinements were carried out with different references. A complex 1 with an indicated global resolution of 2.65 Å at a Fourier shell correlation of 0.143 was generated from one classification of heterogeneous refinement. Another good classification was subjected to multiply heterogeneous refinement with different references. Two complex maps were generated with indicated global resolutions of 2.83 Å and 2.88 Å through 148,610 and 133,068 particles, respectively, and subsequently post-processed by DeepEMhancer.^55^

### Model building and refinement

The initial templates of NTSR1–βArr1 complexes were derived from Swiss-model.^56^ Models were docked into the EM density map using UCSF Chimera^57^ followed by a manual adjustment in Coot.^58^ The model was refined by PHENIX.^59^ The final refinement statistics were validated using the module “comprehensive validation (cryo-EM)” in PHENIX.

### NanoBit-based arrestin recruitment assay

The recruitment of β-arrestin to NTSR1 was detected in HEK293T cells using the NanoLuc Binary System.^23^ The full-length human NTSR1 (wild type or mutants) was cloned into pcDNA3.1 vector with a Flag tag fused at its N-terminus and LgBiT at its C-terminus. Human βArr1 was cloned into pcDNA3.1 vector with a SmBiT fused at its N-terminus. HEK293T cells were grown for 24 h to reach 60%–70% confluence, then transiently transfected with NTSR1-LgBiT and SmBiT-βArr1 at a ratio of 1:1 with Lipofectamine 3000 (Invitrogen) and cultured for another 24 h. The cells were harvested and plated into 96-wells plates at a density of 50,000 cells per well, then reacted with 10 μM Furimazine (TargetMol) for 40 min at room temperature. Luminescence signals were measured for 10 min as baseline using Multifunctional Microplate Reader (FlexStation3, Molecular Devices), and then read for 20 min after addition of ligands. Each mutant was examined in three independent experiments. The data were analyzed using Graphpad Prism software 9.0.

## Supplementary information

Supplementary information, Fig. S1

Supplementary information, Fig. S2

Supplementary information, Fig. S3

Supplementary information, Fig. S4

Supplementary information, Fig. S5

Supplementary information, Fig. S6

Supplementary information, Fig. S7

Supplementary information, Fig. S8

Supplementary information, Fig. S9

Supplementary information, Fig. S10

Supplementary information, Fig. S11

Supplementary information, Table S1

## Data Availability

All data generated or analyzed in this study are included in this article and the supplemental information. The density maps and structure coordinates have been deposited to the Electron Microscopy Data Bank (EMDB) and the Protein Data Bank (PDB) with accession numbers EMD-60579 and 8ZYU for the NTSR1–βArr1 complex 1, EMD-60583 and 8ZYY for the NTSR1–βArr1 complex 2, EMD-60578 and 8ZYT for the NTSR1–βArr1 complex 3, and EMD-63543 and 9M0D for the SBI-553-unbound NTSR1–βArr1 complex.
