# Supplementary material for: Molecular mechanism of the arrestin-biased agonism of neurotensin receptor 1 by an intracellular allosteric modulator
Source: Cell Res. 2025 Mar 21;35(4):284–95. doi: 10.1038/s41422-025-01095-7 (PMC11958688; doi:10.1038/s41422-025-01095-7)
Supplement: Supplementary file 1 — Supplementary information, Fig. S1 [file 41422_2025_1095_MOESM1_ESM.pdf]

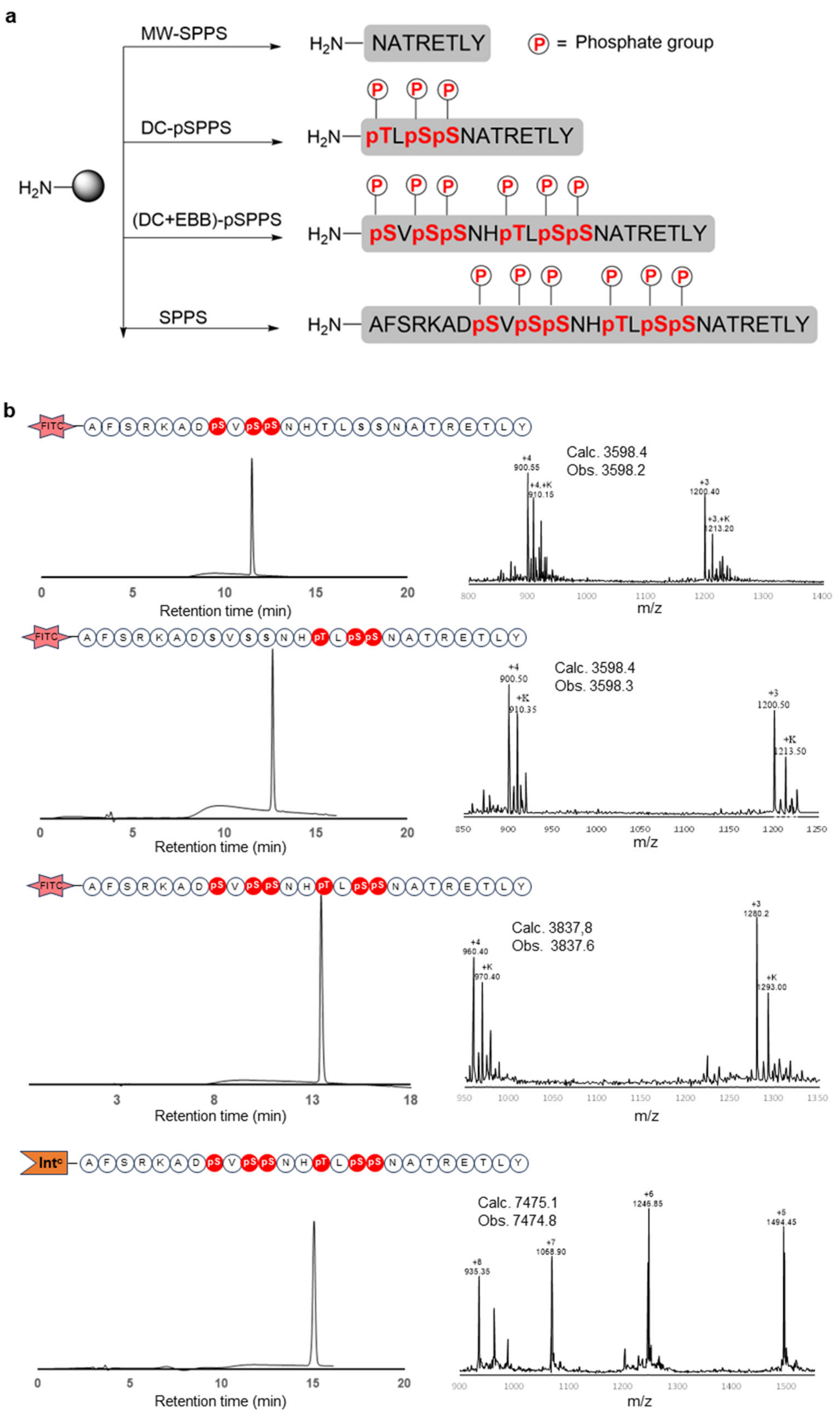

Figure S1. Strategies for the synthesis of multi-phosphorylated peptides. (a) A combination of coupling methods was used according to the specific phosphorylation pattern. *Coupling methods used: MW-solid phase peptide synthesis (SPPS), phospho-SPPS (pSPPS), double coupling-pSPPS (DC-pSPPS) and excess building block pSPPS (EBB-pSPPS). **(b)** HPLC traces and ESI-MS of phosphorylated peptides.
