# Supplementary material for: Molecular mechanism of the arrestin-biased agonism of neurotensin receptor 1 by an intracellular allosteric modulator
Source: Cell Res. 2025 Mar 21;35(4):284–95. doi: 10.1038/s41422-025-01095-7 (PMC11958688; doi:10.1038/s41422-025-01095-7)
Supplement: Supplementary file 2 — Supplementary information, Fig. S2 [file 41422_2025_1095_MOESM2_ESM.pdf]

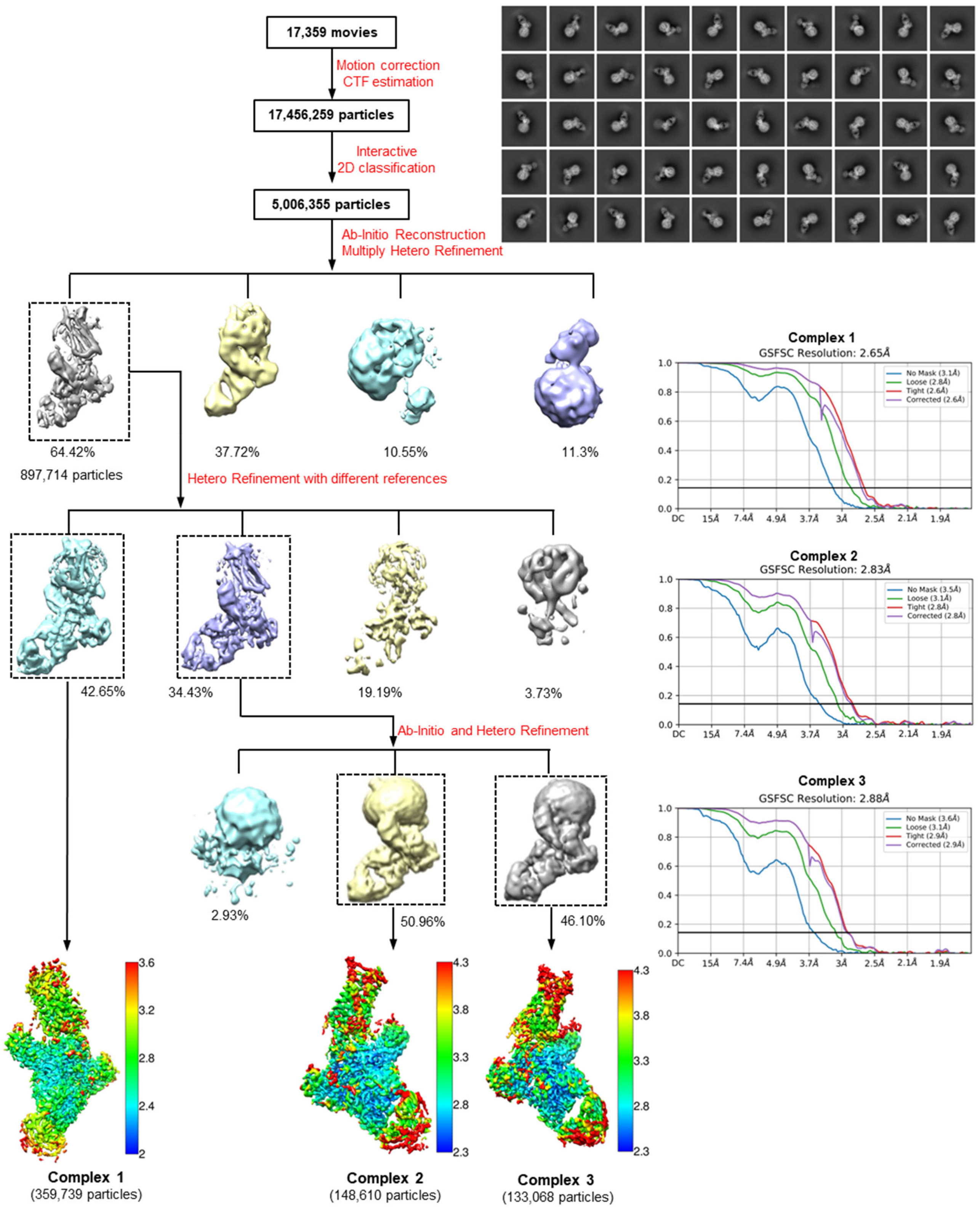

Figure S2. Cryo-EM structure determination of NTSR1-βArr1-SBI-553 complex. Flowchart of cryo-EM data analysis of the NTSR1-βarr1 complex and micrograph of the reference-free 2D class averages are shown. Global Cryo-EM maps of the NTSR1-βarr1 complex complexes were generated and colored by local resolutions from 2 Å (blue) to 4.3 Å (red). The "Gold-standard" Fourier shell correlation (FSC) curve indicates that the resolution of the global electron density map of the NTSR1-βarr1 complex 1, 2 and 3 is 2.65 Å, 2.83 Å and 2.88 Å, respectively.
