# Supplementary material for: Molecular mechanism of the arrestin-biased agonism of neurotensin receptor 1 by an intracellular allosteric modulator
Source: Cell Res. 2025 Mar 21;35(4):284–95. doi: 10.1038/s41422-025-01095-7 (PMC11958688; doi:10.1038/s41422-025-01095-7)
Supplement: Supplementary file 3 — Supplementary information, Fig. S3 [file 41422_2025_1095_MOESM3_ESM.pdf]

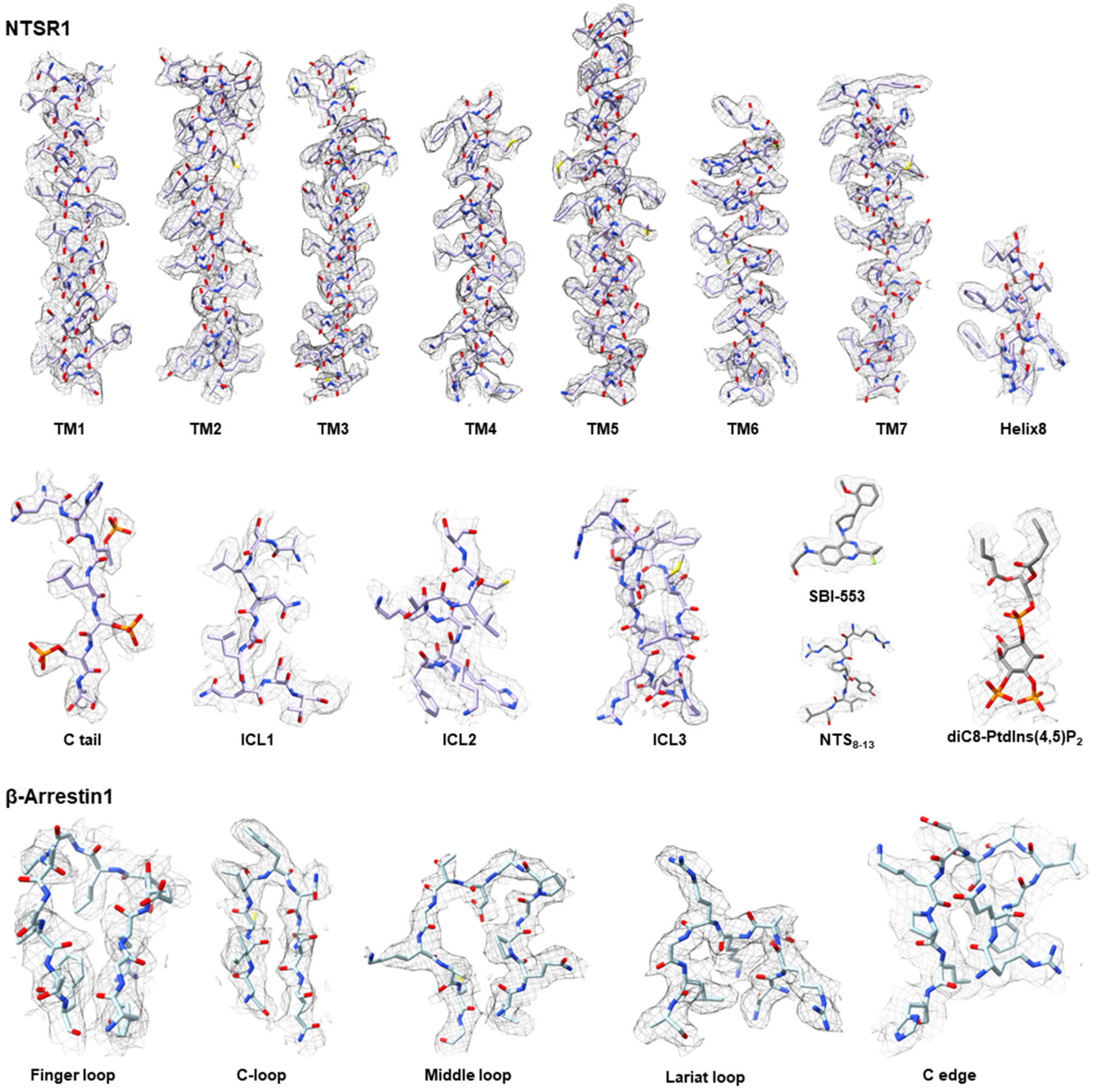

Figure S3. Cryo-EM density analysis of the NTSR1-βArr1-SBI-553 complex 1. Cryo-EM density maps with all transmembrane helices, H8 and intracellular loops of NTSR1 (light purple), loop regions of βArr1 (light blue), NTS, SBI-553 and PIP2 from the NTSR1-βArr1 complex 1.
