# Supplementary material for: Molecular mechanism of the arrestin-biased agonism of neurotensin receptor 1 by an intracellular allosteric modulator
Source: Cell Res. 2025 Mar 21;35(4):284–95. doi: 10.1038/s41422-025-01095-7 (PMC11958688; doi:10.1038/s41422-025-01095-7)
Supplement: Supplementary file 4 — Supplementary information, Fig. S4 [file 41422_2025_1095_MOESM4_ESM.pdf]

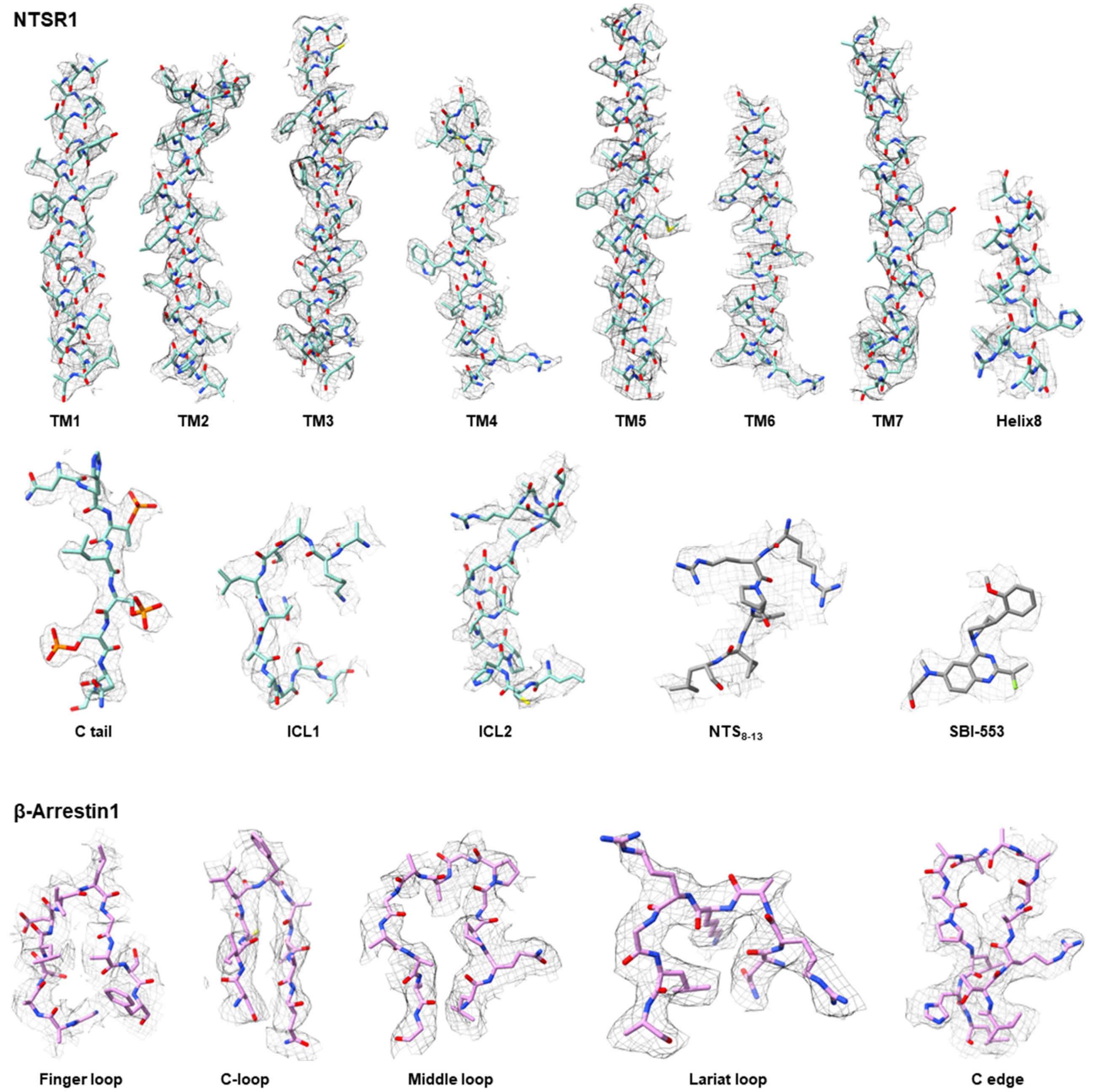

Figure S4. Cryo-EM density analysis of the NTSR1-βArr1-SBI-553 complex 3. Cryo-EM density maps with all transmembrane helices, H8 and intracellular loops of NTSR1 (light cyan), loop regions of βarr1 (light pink), NTS and SBI-553 from the NTSR1-βArr1 complex 3.
