# Supplementary material for: Molecular mechanism of the arrestin-biased agonism of neurotensin receptor 1 by an intracellular allosteric modulator
Source: Cell Res. 2025 Mar 21;35(4):284–95. doi: 10.1038/s41422-025-01095-7 (PMC11958688; doi:10.1038/s41422-025-01095-7)
Supplement: Supplementary file 5 — Supplementary information, Fig. S5 [file 41422_2025_1095_MOESM5_ESM.pdf]

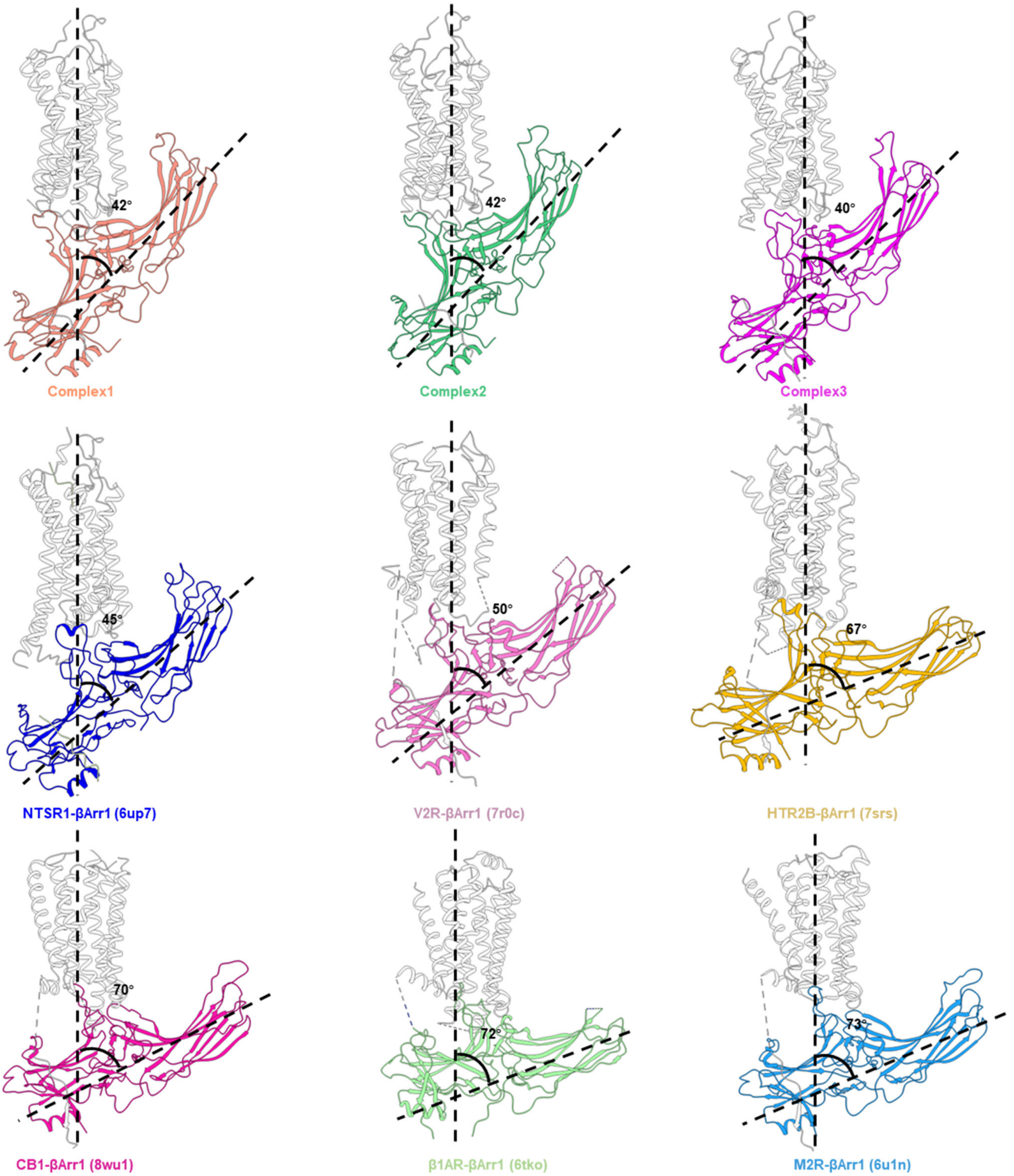

Figure S5. Comparisons of the tilted conformations of βArr1 in the different GPCR complexes. The angle between the longitudinal axis of GPCRs and βArr1 is indicated for each complex.
