# Supplementary material for: Molecular mechanism of the arrestin-biased agonism of neurotensin receptor 1 by an intracellular allosteric modulator
Source: Cell Res. 2025 Mar 21;35(4):284–95. doi: 10.1038/s41422-025-01095-7 (PMC11958688; doi:10.1038/s41422-025-01095-7)
Supplement: Supplementary file 6 — Supplementary information, Fig. S6 [file 41422_2025_1095_MOESM6_ESM.pdf]

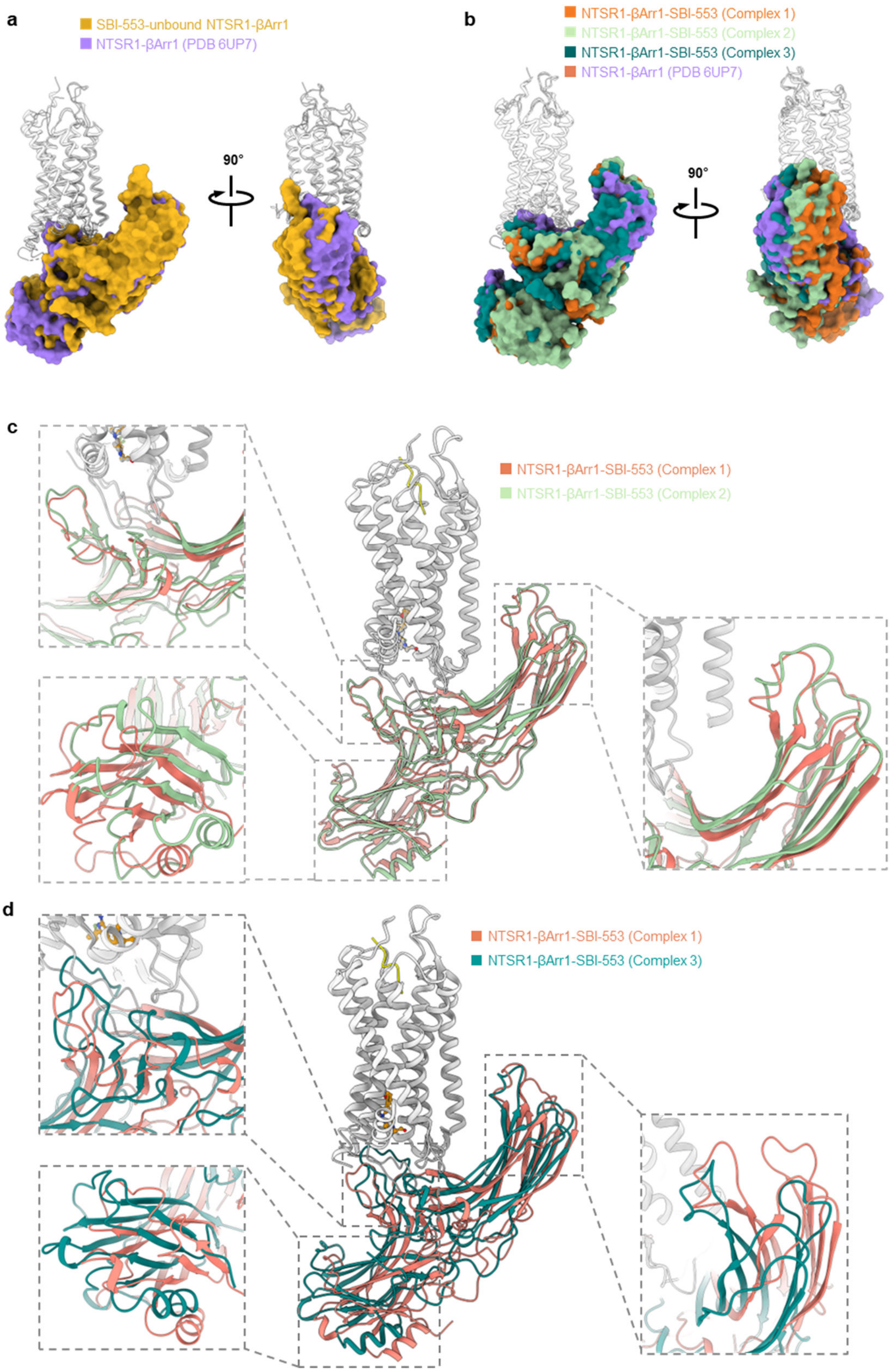

Figure S6. Structural comparison of the NTSR1-βArr1 complexes. (a, b) Overlay of the SBI-553-unbound NTSR1-βArr1 complex (a) and SBI-553-bound NTSR1-βArr1 complex 1, 2 and 3 structures (b) with previously reported NTSR1-βArr1 structure (PDB: 6UP7) on the basis of alignment of the receptor chains. **(c, d)** Overlay of the NTSR1-βArr1 complex 1 and 2 structures (b), the NTSR1-βArr1 complex 1 and 3 structures (c) on the basis of alignment of the receptor chains.
