# Supplementary material for: Molecular mechanism of the arrestin-biased agonism of neurotensin receptor 1 by an intracellular allosteric modulator
Source: Cell Res. 2025 Mar 21;35(4):284–95. doi: 10.1038/s41422-025-01095-7 (PMC11958688; doi:10.1038/s41422-025-01095-7)
Supplement: Supplementary file 7 — Supplementary information, Fig. S7 [file 41422_2025_1095_MOESM7_ESM.pdf]

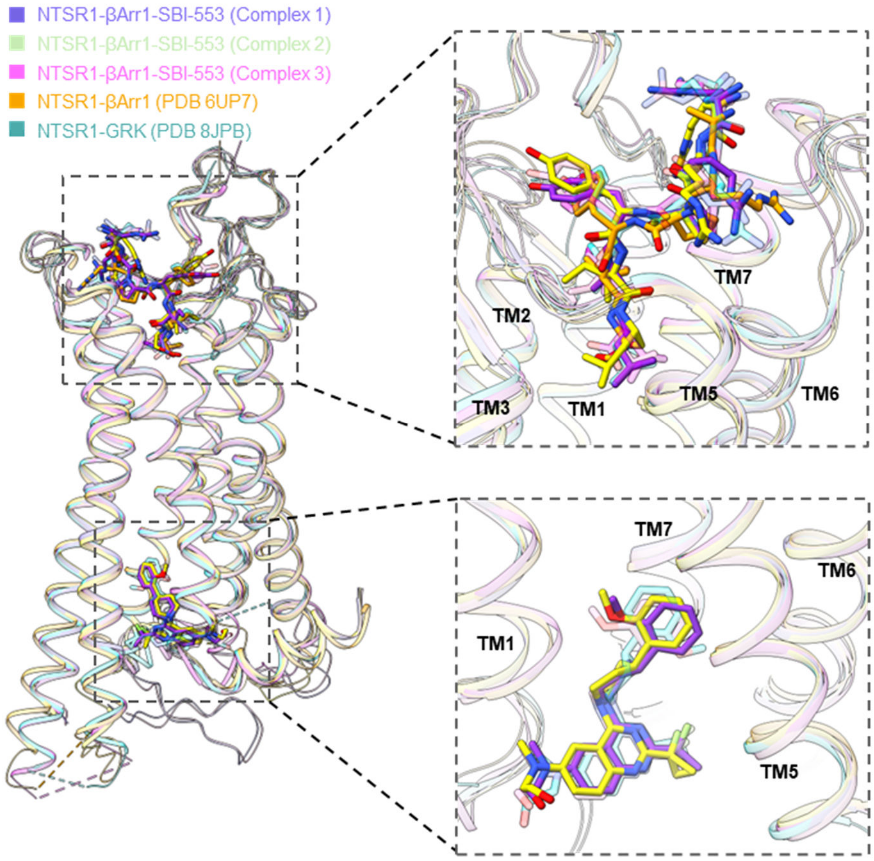

Figure S7. The binding of NTS peptide and SBI-553 in NTSR1-βArr1-SBI-553 complex. Superposition of the receptor TMD fragment from different GPCR complexes showed that the receptors have very similar 3D architecture, and the location of NTS peptide and SBI-553 from different G proteins overlapped.
