# Supplementary material for: Molecular mechanism of the arrestin-biased agonism of neurotensin receptor 1 by an intracellular allosteric modulator
Source: Cell Res. 2025 Mar 21;35(4):284–95. doi: 10.1038/s41422-025-01095-7 (PMC11958688; doi:10.1038/s41422-025-01095-7)
Supplement: Supplementary file 8 — Supplementary information, Fig. S8 [file 41422_2025_1095_MOESM8_ESM.pdf]

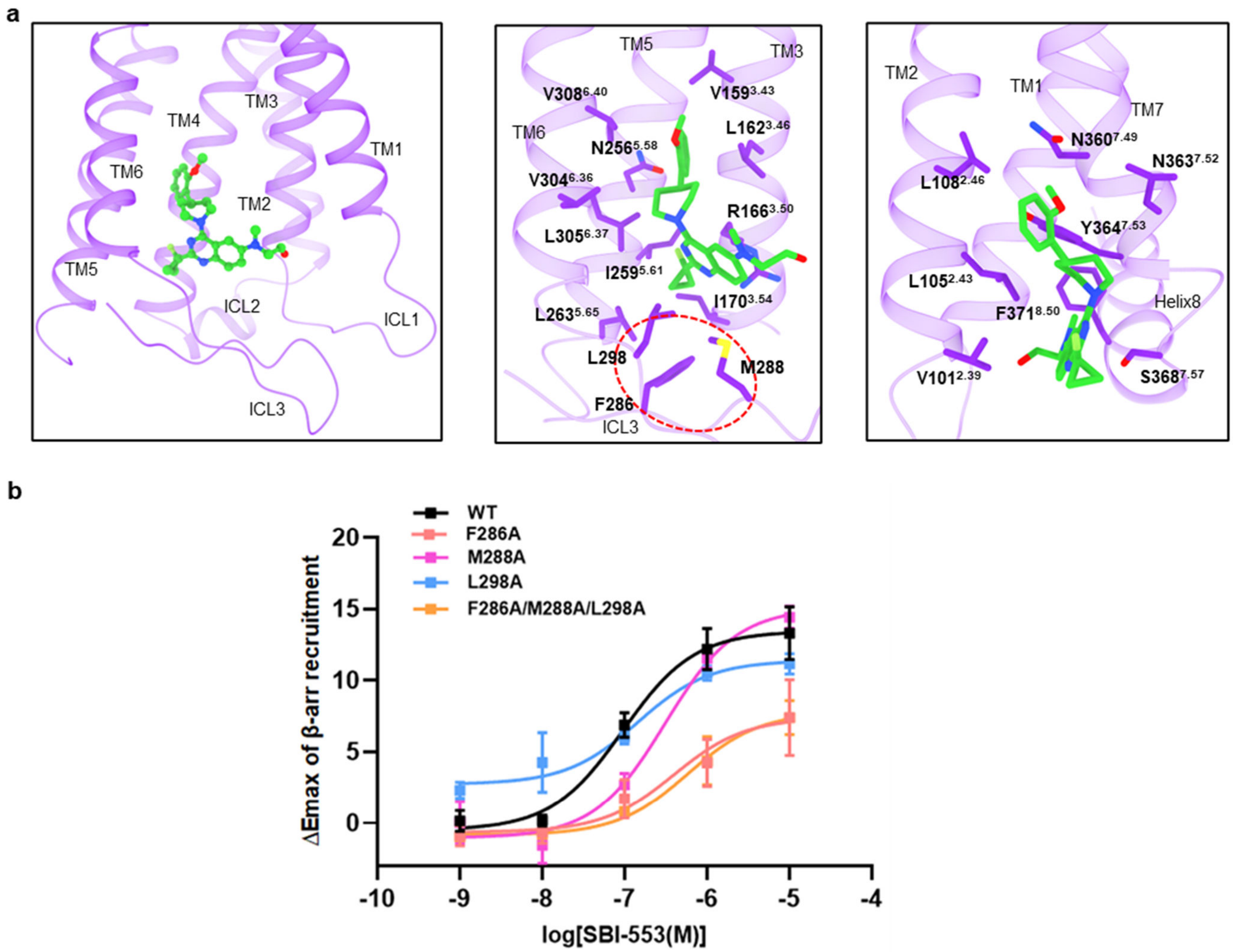

	WT	F286A	M288A	L298A	F286A/M288A/L298A
EC_{50}	9.48e-008	3.94e-007	3.12e-007	1.44e-007	5.87e-007
Emax	13.95%	8.03%	8.66%	16.13%	8.63%

Figure S8. The binding SBI-553 in NTSR1-βArr1-SBI-553 complex. (a) Detailed interactions between SBI-553 and NTSR1 in the NTSR1-βArr1-SBI-553 complex 1. NTSR1 is shown in purple and SBI-553 is shown in green. **(b)** Mutation-based arrestin recruitment assay to verify the interactions between ICL3 and SBI-553. ΔEmax=Emax(NTS+SBI-553)-Emax(NTS). Data are mean ± s.d. from at least three independent experiments (n ≥ 3).
