# Supplementary material for: Molecular mechanism of the arrestin-biased agonism of neurotensin receptor 1 by an intracellular allosteric modulator
Source: Cell Res. 2025 Mar 21;35(4):284–95. doi: 10.1038/s41422-025-01095-7 (PMC11958688; doi:10.1038/s41422-025-01095-7)
Supplement: Supplementary file 9 — Supplementary information, Fig. S9 [file 41422_2025_1095_MOESM9_ESM.pdf]

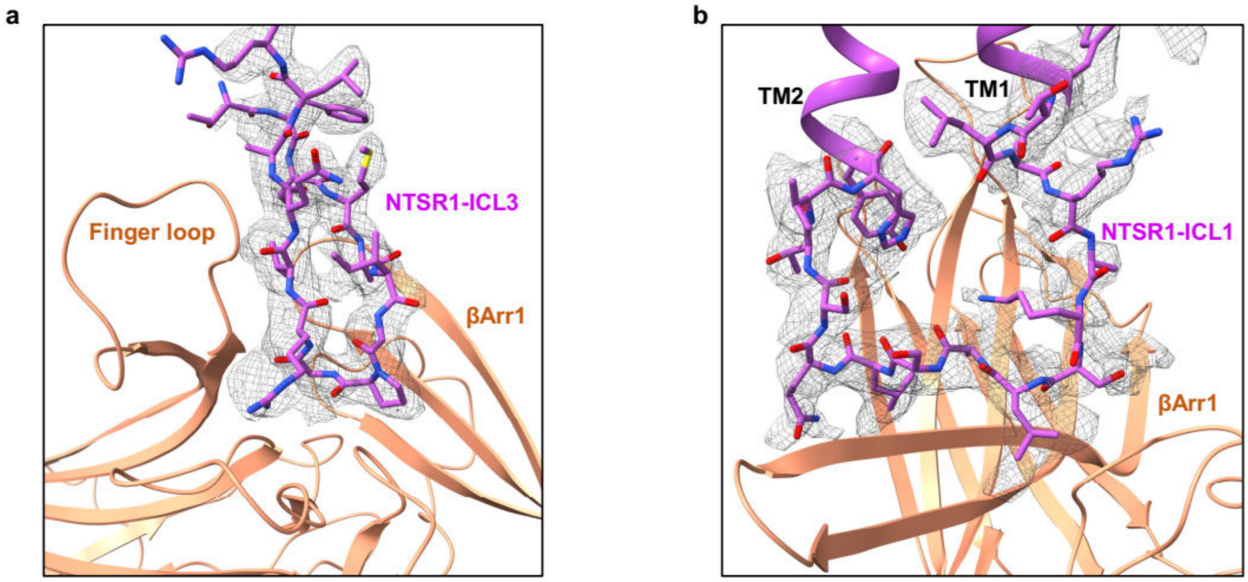


**Figure S9. The interactions between NTSR1 ICLs and βArr1 in NTSR1-βArr1-SBI-553 complex.** Density maps of ICL3 (a) and ICL1 (b) are presented.
