# Supplementary material for: Molecular mechanism of the arrestin-biased agonism of neurotensin receptor 1 by an intracellular allosteric modulator
Source: Cell Res. 2025 Mar 21;35(4):284–95. doi: 10.1038/s41422-025-01095-7 (PMC11958688; doi:10.1038/s41422-025-01095-7)
Supplement: Supplementary file 10 — Supplementary information, Fig. S10 [file 41422_2025_1095_MOESM10_ESM.pdf]

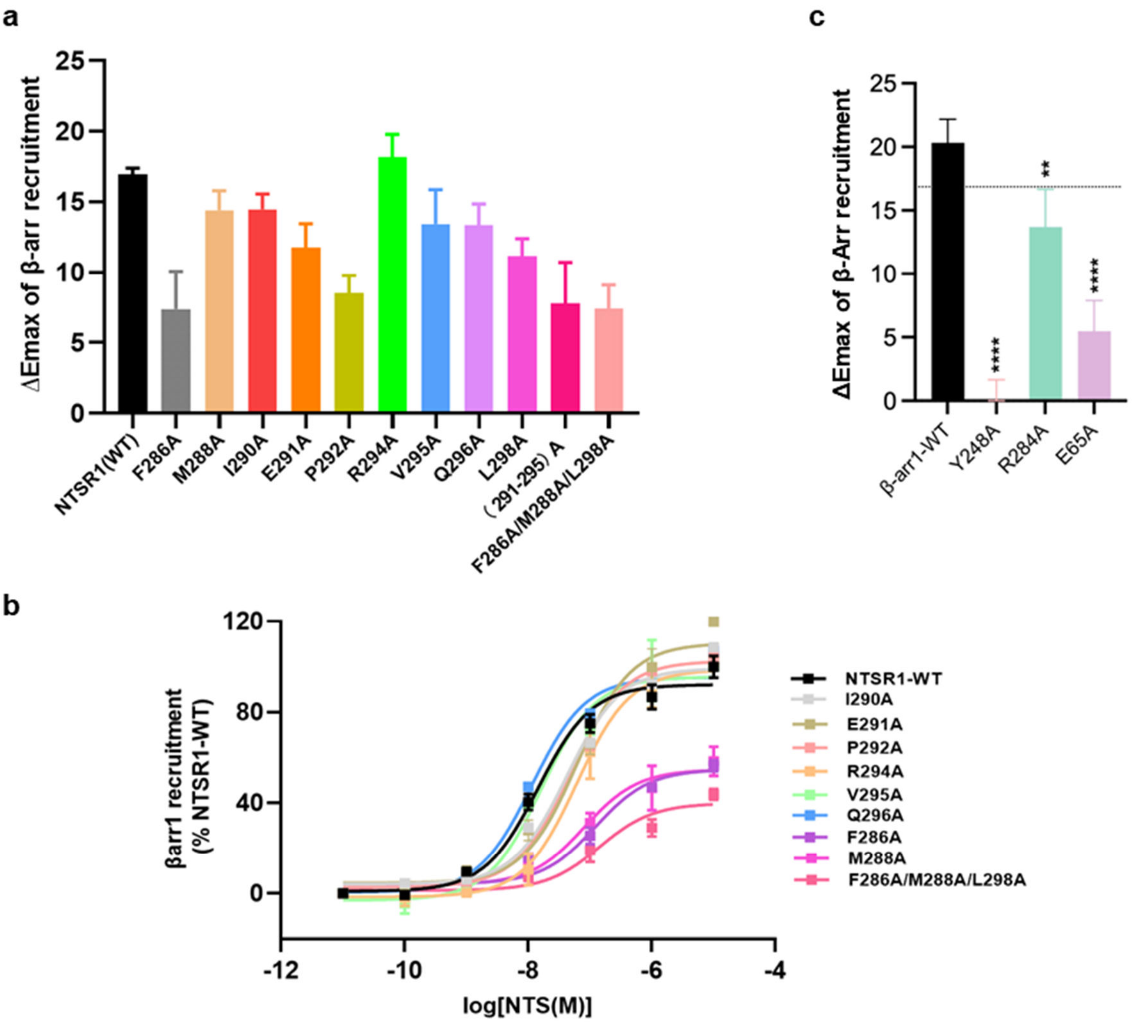

	WT	I290A	E291A	P292A	R294A	V295A	Q296A	F286A	M288A	F286A/M288A/L298A
EC50	1.444e-008	4.189e-008	6.232e-008	4.694e-008	6.101e-008	1.633e-008	1.151e-008	1.232e-007	8.119e-008	1.434e-007
Emax	100	108.62	119.85	104.40	102.24	100.11	107.06	56.34	58.29	43.76

Figure S10. βArr1 recruitment to NTSR1 induced by NTS and SBI-553. (a) Recruitment of βArr1 to wild-type (WT) and mutated NTSR1 induced by NTS (1 nM) and SBI-553 (10 μM). △Emax=Emax(NTS+SBI-553)-Emax(NTS). Data are mean ± s.d. from three independent experiments (n = 3). **(b)** The βArr1 recruitment effects of the mutations on NTSR1 mutants stimulated only by NTS. **(c)** Recruitment of mutated βarr1 to wild-type NTSR1 induced by NTS (1 nM) and SBI-553 (10 μM). △Emax=Emax(NTS+SBI-553)-Emax(NTS). Data are mean ± s.d. from at least three independent experiments (n ≥ 3).
