# Supplementary material for: Molecular mechanism of the arrestin-biased agonism of neurotensin receptor 1 by an intracellular allosteric modulator
Source: Cell Res. 2025 Mar 21;35(4):284–95. doi: 10.1038/s41422-025-01095-7 (PMC11958688; doi:10.1038/s41422-025-01095-7)
Supplement: Supplementary file 11 — Supplementary information, Fig. S11 [file 41422_2025_1095_MOESM11_ESM.pdf]

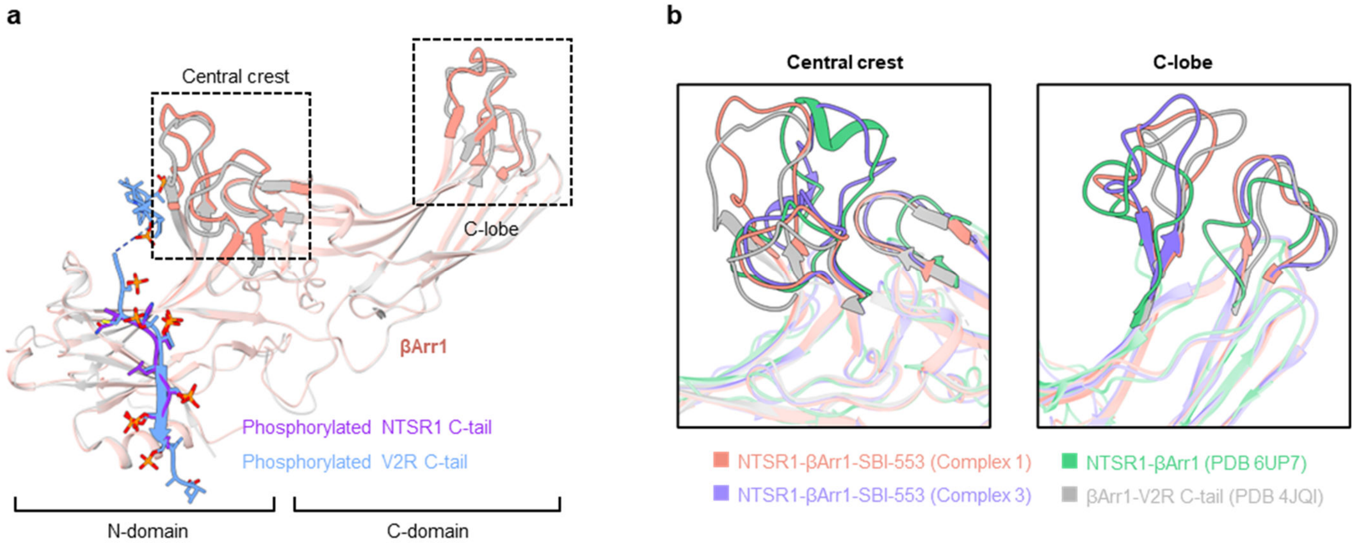

Figure S11. Structural comparisons of the conformational change of βArr1 in different complexes. (a) The βArr1 in the NTSR1-βArr1-SBI-553 complex 1 is superimposed onto the βArr1 in the complex of βArr1-V2R C-tail peptide. The phosphorylated NTSR1 C-tail peptide and V2R C-tail peptide are show in violet and blue respectively. The Phosphorylate groups in the two peptides are shown in sticks. **(b)** Structural alignment of arrestin shows various configurations of the loops in the central crest and C-lobe when binding to different GPCRs.
