# Supplementary material for: Molecular mechanism of the arrestin-biased agonism of neurotensin receptor 1 by an intracellular allosteric modulator
Source: Cell Res. 2025 Mar 21;35(4):284–95. doi: 10.1038/s41422-025-01095-7 (PMC11958688; doi:10.1038/s41422-025-01095-7)
Supplement: Supplementary file 12 — Supplementary information, Table S1 [file 41422_2025_1095_MOESM12_ESM.pdf]

Table S1. Cryo-EM data collection, refinement and validation statistics.

	SBI-553-bound Complex1	SBI-553-bound Complex2	SBI-553-bound Complex3	SBI-553-unbound complex
**Data collection and processing**				
Magnification	105,000	105,000	105,000	105,000
Voltage (kV)	300	300	300	300
Electron exposure (e^-/Å²)	50	50	50	50
Defocus range (μm)	-1.2 to -1.8	-1.2 to -1.8	-1.2 to -1.8	-1.4 to -2.2
Pixel size (Å)	0.824	0.824	0.824	0.824
Symmetry imposed	C1	C1	C1	C1
Initial particle images (no.)	17,456,259	17,456,259	17,456,259	6,359,592
Final particle images (no.)	359,739	148,610	133,068	156,997
Map resolution (Å)				
FSC threshold	0.143	0.143	0.143	0.143
Map resolution (Å)	2.65	2.83	2.88	3.41
**Structure Refinement**				
Model resolution (Å)	2.8	3.3	3.1	3.7
FSC threshold	0.5	0.5	0.5	0.5
Model-Map CC (mask)	0.8	0.76	0.75	0.75
Model composition				
Non-hydrogen atoms	8,641	7,763	8,425	6313
Protein residues	1,128	1,112	1,127	1044
B factors (Å²)				
Protein	50.23	78.34	76.31	41.49
Ligands	57.76	85.75	68.24	/
R.m.s. deviations				
Bond lengths (Å)	0.003	0.002	0.003	0.005
Bond angles (°)	0.589	0.515	0.530	0.682
**Validation**				
MolProbity score	1.68	1.54	1.60	1.76
Clash score	7.92	5.40	6.33	6.56
Rotamer outliers (%)	0.22	0	0.23	0.58
Ramachandran plot				
Favored (%)	96.30	96.34	96.29	94.01
Allowed (%)	3.70	3.66	3.71	5.99
Disallowed (%)	0	0	0	0
